# PRR11 promotes cell proliferation by regulating PTTG1 through interacting with E2F1 transcription factor in pan-cancer

**DOI:** 10.3389/fmolb.2022.877320

**Published:** 2022-08-19

**Authors:** Haibo Zhang, Ziqing He, Li Qiu, Jinfen Wei, Xiaocheng Gong, Mingjian Xian, Zixi Chen, Ying Cui, Shuying Fu, Zihao Zhang, Bowen Hu, Xiquan Zhang, Shudai Lin, Hongli Du

**Affiliations:** ^1^ School of Biology and Biological Engineering, South China University of Technology, Guangzhou, China; ^2^ College of Animal Science, South China Agricultural University, Guangzhou, China; ^3^ College of Coastal Agricultural Sciences, Guangdong Ocean University, Zhanjiang, GD, China

**Keywords:** proline rich 11 (PRR11), pituitary tumor-transforming gene 1 (PTTG1), cell cycle, cell migration, pan-cancer

## Abstract

The upregulated proline rich 11 (PRR11) plays a critical role in cancer progression. The relevant biological functions of PRR11 in pan-cancer development are not well understood. In the current study, we found that *PRR11* was upregulated in 19 cancer types compared with that of normal tissues and high-expressed *PRR11* was a predictor of poor prognosis in 10 cancer types by bioinformatics. Then we showed that interfering PRR11 on three cancer cell lines could greatly inhibit cell proliferation and migration and arrest cells to S phase *in vivo*. Based on RNA-seq, downregulation of *PRR11* expression could extremely suppress the expression of *PTTG1* and the cell cycle pathway identified by a differentially expressed gene analysis and an enrichment analysis. The expression of *PRR11* and *PTTG1* was positively correlated in TCGA and independent GEO data sets. Importantly, we revealed that the PRR11 could express itself in the nucleus and interact with E2F1 on the *PTTG1* promoter region to increase the expression of *PTTG1*. Further results indicated that the expression of *PTTG1* was also associated with poor prognosis in 10 cancer types, while downregulation of *PTTG1* expression could inhibit cancer cell proliferation and migration. Therefore, we found that PRR11 served as an oncogene in pan-cancer and could influence the cell cycle progression through regulating the expression of *PTTG1* by interacting with the transcription factor E2F1.

## Introduction

Studies have shown that proline rich 11 (PRR11) contains a bivalent nuclear localization signal, two proline-rich regions, and a zinc finger domain, which is a well-known domain that can bind double-stranded DNA, modulate gene transcription (Zhou et al., 2019), and participate in cell signal transduction leading to a series of cancer-related events (Qiao et al., 2019).

Previous studies have suggested that PRR11 plays a vital role in numerous biological processes, such as cell cycle progression; cell proliferation, migration, and invasion; apoptosis; and cell autophagy of tumor cells by regulating cyclin genes (Ji et al., 2013; Zhang et al., 2018), p38 MAPK (Lin et al., 2020), Wnt/β-catenin (Qiao et al., 2019; Zhou et al., 2019), PI3K/AKT/β-catenin (Zhu et al., 2018), and Akt/mTOR autophagy (Zhang et al., 2018) signaling pathways. Many cyclins and cyclin-related genes, including *CCNA1*, *RRM1*, *PCNA* (Ji et al., 2013), *CDK6*, *CCNE*, *CCNA2*, and *CCNB2*, were inhibited when interfering with PRR11 (Zhang et al., 2018). In addition, the low-expressed PRR11 could also suppress the expression level of apoptotic proteins, such as P62 (Zhang et al., 2018). However, the precise molecular mechanisms underlying how PRR11 regulates the expression of genes involved in different signaling pathways and whether high-expressed *PRR11* has a common regulatory mechanism in the development of different cancer types are yet to be elucidated. Therefore, finding out the molecular regulation mechanisms of *PRR11* upregulation in promoting tumorigenesis and development of pan-cancers will help us understand its function and develop new diagnostic and therapeutic methods.

In this study, we examined the expression pattern of *PRR11* in different types of cancers with ≥ 30 normal tissues from TCGA and explored its related clinical pathological, to clarify the application prospect of PRR11 as a prognostic marker of pan-cancers. In addition, we revealed the related regulation pathways by conjoint analyzing the differentially expressed genes (DEGs) from RNA-seq data of cell lines and TCGA datasets and evaluated the biological function and the molecular mechanism of PRR11 in pan-cancer development using ChIP-qPCR, Western blot, and a series of experimental techniques. Mechanically, *PRR11* could regulate the expression of *PTTG1* by interacting with E2F1 to the E2F1 binding site of the *PTTG1* promoter region. These results demonstrated that *PRR11* could play a key role during the development and progression of pan-cancer and might represent a novel and valuable prognostic marker and therapeutic target for pan-cancer progression.

## Materials and methods

### Data source

Expression data and corresponding clinical information of different kinds of cancer patients were downloaded from The Cancer Genome Atlas (TCGA, https://portal.gdc.cancer.gov/). The GSE48075 set was downloaded from the Gene Expression Omnibus database (GEO, http://www.ncbi.nlm.nih.gov/geo/) and used to validate interested genes overall survival of patients.

### Prognostic analysis of patients in pan-cancer

The clinical outcome of patients with different types of cancers was determined using the Kaplan–Meier survival curves. For the overall survival, the samples were divided into two groups according to the median expression of the mRNAs (high vs low) with the use of R packages (survival, version 3.2.7; survminer, version 0.4.8). The *p* < 0.05 indicated statistically significant differences.

### Identification of DEGs of pan-cancers from TCGA

The samples of 11 types of cancers, which were *PRR11*, were remarkably upregulated and with normal tissues ≥ 30 in TCGA databases. The expression levels of *PRR11* in all individuals in each kind of 11 types of cancers were ranked from high to low and then separated samples into 30% PRR11 high and 30% low groups to find out DEGs using the “DESeq2” package (version 1.28.1) in R language (version 4.0.2) (Love et al., 2014). |Fold Change| > 1.5 and FDR < 0.05 were set as the statistical threshold value of DEGs.

### Functional enrichment analysis

The Gene Ontology (GO) and Kyoto Encyclopedia of Genes and Genomes (KEGG) analyses were conducted by using the R package (clusterProfiler, version 3.16.1) to explore different molecular mechanisms and involved pathways between PRR11 high and low patients. The number of permutations was set at 1,000, and the cutoff *P*-adjust was 0.05 to filter the significant enrichment results. The protein–protein interaction (PPI) network of DEGs was obtained from the STRING (version 11.0) database (Szklarczyk et al., 2019). We used Cytoscape (version 3.5.1) software to visualize the PPI network and loaded the plug-in Molecular Complex Detection (MCODE) for a sub-network analysis, followed by extracting the densely connected areas in the PPI network, and built key modules (Bader and Hogue, 2003).

### Spearman correlation between *PRR11* and *PTTG1*

Spearman’s correlation coefficient analysis was performed to explore the correlation between *PRR11* and *PTTG1* in different cancers that ≥ 200 tumor tissues from the TCGA database.

### Cell culture

The liver, lung, and pancreatic cancer cell lines (HepG2, NCI-H460, and BxPC3) were gifts from Dr. Peng Wang, Sun Yat-sen University Cancer Hospital, Guangzhou, China. HepG2 was isolated from a hepatocellular carcinoma of a 15-year-old, white, male with liver cancer. NCI-H460 cells were isolated from the pleural fluid of a male patient with large-cell lung cancer. In addition, BxPC3 was isolated from the pancreas tissue of a 61-year-old female patient with adenocarcinoma. HepG2 cells were cultured in DMEM/high glucose, and NCI-H460 and BxPC3 cells were cultured in the RPMI-1640 basic medium, separately supplemented with 10% fetal bovine serum (Gibco, NY, United States), 100 U/ml penicillin, and 100 μg/ml streptomycin at 37°C in air with 5% CO_2_.

### RNA interference and transfection

The specific siRNAs of *PRR11*, *E2F1*, and *PTTG1* genes and a nonsense siRNA were synthesized and purified by Hanheng (Shanghai, China) (Supplementary Table S1). A total of 5 × 10^4^, 2 × 10^4^, and 3 × 10^3^ cells per well were seeded in 6-well, 12-well, and 96-well plates, respectively. After 24 h, cells were transfected with siRNAs as 40 nM/ml (Supplementary Figure S1) using Lipofectamine 3000 (Thermo Fisher Scientific, Waltham, MA, United States), according to the manufacturer’s protocol.

### RNA-seq

Total RNA was extracted from the cells using the TRIzol reagent (Thermo Fisher Scientific, Waltham, MA, United States) and kept at −80°C. The complementary DNA (cDNA) libraries of each pooled RNA sample for single-end sequencing were generated using the NEBNext^®^ UltraTM RNA Library Prep Kit for Illumina^®^ (NEB, Cat. No. E7530L, New England Biolabs, United States), according to the manufacturer’s instructions. The cDNA libraries were subjected to the NovaSeq 6,000 system (Illumina, CA, United States), according to commercially available protocols. The changed RNAs were validated by quantitative PCR using the primers listed in Supplementary Table S2.

### RNA-seq analysis

The raw sequencing data were evaluated by FAST-QC, while the transcriptional start site (TSS) and chromosomal distribution were obtained by custom java scripts. The potential genes were defined within +5 kilobases (kb) from the TSS and 50 kb downstream from the transcription end site. The clean reads from RNA-seq were aligned to the human reference genome sequence GRCH38.p13 using the HISAT2 program (v2.2.3) (Kim et al., 2019). The experimental data were first optimized for the alignment parameters to provide the largest information on the AS events. The potential genes sequenced were counted by HTseq and their relative expression levels were determined by GENCODE (v36) (Frankish et al., 2019).

### Quantitative real-time PCR

Total RNA was reverse transcribed into cDNA by using the PrimeScript RT-polymerase (TaKaRa, Bao Biological Engineering (Dalian) Co., Ltd. China). Quantitative real-time PCR was performed on the cDNA templates using specific primers (Sangon Biotech, Shanghai, China) (Supplementary Table S2) and SYBR master mix (TaKaRa, Bao Biological Engineering (Dalian) Co., Ltd. China), according to the manufacturer’s protocols. The relative mRNA expression levels of interested genes were calculated as a ratio normalized to *GAPDH* expression. Comparative quantification was performed using the 2^−ΔΔCt^ method.

### Cell cycle analysis

After 48-h treatment of siNC and siPRR11-2, cells were washed twice with pre-cooled 1X phosphate-buffered saline (PBS) (Gibco, NY, United States), centrifuged at 1,200 rpm 4°C for 5 min, fixed with 70% ethanol, and then stained with propidium iodide, according to an established protocol. Then the cells’ cell cycle was analyzed on a BD FACS Calibur (Becton, Dickinson and Company, United States).

### Cell proliferation and wound healing assays

The influence of PRR11-specific siRNA on cell viability was first tested using a glass hemocytometer and coverslip. In brief, cells growing in the log phase were trypsinized and seeded in 6-well plates (2–6 × 10^5^ cells per well in a final volume of 2 ml) in four replicates and incubated at 37°C in air with 5%. After incubation for 0, 24, 48, 72, or 96 h, 200 µl of 0.25% trypsin (Gibco, NY, United States) was added per well to digest the cells, stop the digestion with 1.8 ml of complete cell cultural medium, and collect them in an Eppendorf tube. Furthermore, we took 50 ul of cell suspension into a new Eppendorf tube, added 50 ul 0.4% Trypan Blue (final concentration 0.2%), and mixed gently. We filled both chambers of glass hemocytometer underneath the coverslip and took 10 ul of trypan blue–treated cell suspension and applied to the hemocytometer. Using a microscope, we focused on the grid lines of the hemocytometer with a ×10 objective. We counted the live, unstained cells in all four sets of 16 squares using a hand tally counter. Finally, we calculated the number of viable cells per well: Number of cells = (the cell count from four sets of 16 squares/4) × 10^4^ × 2 (dilution factor) × 2 (volume).

The effect of PRR11-specific siRNA on cell viability was tested using a CCK-8 assay kit (Beyotime Biotechnology, Shanghai, China), according to the manufacturer’s instructions. In brief, cells growing in the log phase were trypsinized and seeded in 96-well plates (3,000 cells per well in a final volume of 100 µl) in four to six replicates and incubated at 37°C in air with 5% CO_2_. After incubation for 0, 24, 48, 72, or 96 h, 10 µl of CCK-8 was added to each well and incubated at 37°C for an additional 1 h. The quantity of formazan product was measured by its absorbance at 450 nm using a 96-well plate reader (Molecular Devices). All experiments were repeated at least three times.

A wound healing assay was performed to detect the migration of three kinds of cancer cell lines. Cells growing in the log phase were trypsinized and seeded in 24-well plates until confluence. The cell layer was transfected with siPRR11-2 and wounded using a sterile tip. After incubation for 0, 24, 48, 72, and 96 h, cells were photographed under an inverted microscope, respectively. The distance between the two edges of the scratch (wound width) was measured at eight sites using ImageJ in each image (×40 magnification).

### Cellular immunofluorescence

A total of 10^4^ NCI-H460 cells were observed and pictures were taken every 24 h using a laser confocal microscope; washed twice with PBS after 48 h, 1 ml each time; and shook slowly for 3 min on a shaker. Then they were fixed with 1 ml of 4% paraformaldehyde for 30 min, washed with 1 ml PBS, and shook slowly for 5 min for three times, followed by 0.1% Triton X-100 (prepared in PBS) permeation for 10 min at room temperature, and washed three times with PBS as above. Furthermore, we added 1 ml of Quick-block solution (Beyotime Biotechnology, P0235, Shanghai, China) for 30 min at room temperature, washed three times with PBS as above, then removed the blocking solution, added 1 ml of diluted PRR11 primary antibody (Thermo Fisher Scientific, 1:1,000, Cat. No. PA5113175, Waltham, MA, United States), and incubated overnight at 4°C. Then we washed the cells with PBS three times for 5 min each time, added the diluted fluorescent secondary antibody (Thermo Fisher Scientific, 1:5,000, Cat. No. A11012, Waltham, MA, United States), incubated for 1 h at room temperature in the dark, followed by washing three times with PBS for 3 min each time. Then cells were stained with nucleus DAPI dropwise, incubated for 5 min in the dark, and washed four times with PBS for 5 min. We dropped the mounting solution containing an anti-fluorescence quencher (Thermo Fisher Scientific, Cat. No. MAN0010261, Waltham, MA, United States) and then observed and photographed the image under a fluorescence microscope (Leica TCS SP8 X, Germany).

### Chromatin immunoprecipitation

Chromatin immunoprecipitation (ChIP) was performed according to the instructions of the Pierce Agarose ChIP Kit (Thermo Fisher Scientific, Cat. No. 26156, Waltham, MA, United States). The E2F1 binding site on the *PTTG1* promoter region (NC_000005.10, 1,60419855–160421854) was predicted using the JASPAR online tool (http://jaspar.genereg.net/). A fluorescence quantitative PCR was carried out using *PTTG1* promoter specific primers (Supplementary Table S2). The relative expression levels of interested *PTTG1* promoter regions were calculated as a ratio normalized to *GAPDH* (primers are provided by the ChIP kit) expression when *E2F1* was knocked down and negative control for 48 h in NCI-H460 cells. A comparative quantification was performed using the 2^−ΔΔCt^ method.

### Co-immunoprecipitation and Western blotting

Co-immunoprecipitation (Co-IP) was performed according to the instructions of the Pierce™ Classic Magnetic IP/Co-IP Kit (Thermo Fisher Scientific, Cat. No. 88804, Waltham, MA, United States). Proteins were quantified and resolved by 10% SDS-PAGE and electrotransferred to polyvinylidene difluoride (PVDF) membranes (Millipore, Bedford, MA, United States) followed by blocking in Quick-block solution (Beyotime Biotechnology, Shanghai, China) at room temperature for 30 min and incubation with primary antibodies against PRR11 (Thermo Fisher Scientific, 1:200, Cat. No. PA5113175, Waltham, MA, United States) and E2F1 (Thermo Fisher Scientific, 1:2,000, Cat. No. MA123202, Waltham, MA, United States) overnight at 4°C. Then the PVDF membranes were incubated with IgG horseradish peroxidase (HRP)–conjugated secondary antibodies (Beyotime Biotechnology, Cat. No. A0208 and A0216, Shanghai, China) and detected using the electrochemiluminescence (ECL) (Beyotime Biotechnology, Cat No. P0018FS, Shanghai, China) method, according to the manufacturer’s instructions. The group without any antibody was used as a mock control.

### Statistical analysis

The analyses were carried out using “R” software (version 4.0.2) with corresponding packages and IBM SPSS Statistics 19. Error bars in graphs represent ±standard error of mean of more than triplicate values, and statistical significance was determined by Student’s t-test when only two groups were compared. The *p* value < 0.05 was considered statistically significant.

## Results

### The high-expressed *PRR11* was significantly associated with poor overall survival of TCGA cohort patients

**FIGURE 1:**
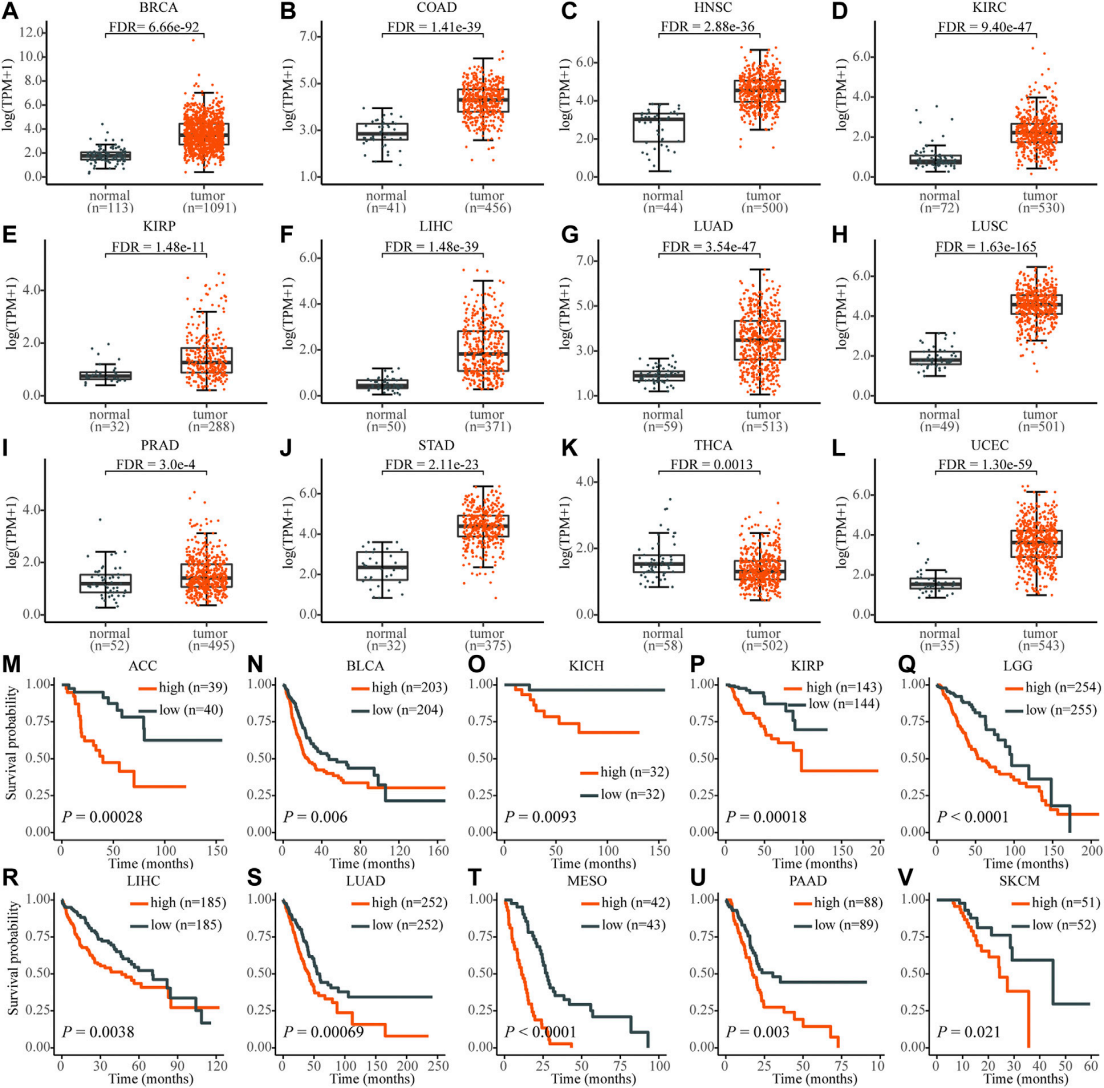
The expression level of PRR11 of normal tissues (black) and tumor tissues (red) in pan-cancer and its high expression are significantly associated with poor prognosis. **(A)** BRCA, breast invasive carcinoma; **(B)** COAD, colon adenocarcinoma; **(C)** HNSC, head and neck squamous cell carcinoma; **(D)** KIRC, kidney renal clear cell carcinoma; **(E)** KIRP, kidney renal papillary cell carcinoma; **(F)** LIHC, liver hepatocellular carcinoma; **(G)** LUAD, lung adenocarcinoma; **(H)** LUSC, lung squamous cell carcinoma; **(I)** PRAD, prostate adenocarcinoma; **(J)** STAD, stomach adenocarcinoma; **(K)** THCA, thyroid carcinoma; **(L)** UCEC, uterine corpus endometrial carcinoma; **(M)** ACC, adrenocortical carcinoma; **(N)** BLCA, bladder urothelial carcinoma; **(O)** KICH, kidney chromophobe; **(P)** KIRP; **(Q)** LGG, brain lower grade glioma; **(R)** LIHC; **(S)** LUAD; **(T)** MESO, mesothelioma; **(U)** PAAD, pancreatic adenocarcinoma; **(V)** SKCM, skin cutaneous melanoma.

In total, 19 out of 33 cancer tissues were PRR11 upregulated in the TCGA database, compared with that of normal tissues (Figures 1A–L and Supplementary Figure S2. From the result of cancer tissue samples with corresponding normal tissue samples ≥ 30, except for thyroid carcinoma (THCA), the expression of PRR11 in the tumor tissues was significantly higher than that in normal tissues (FDR<0.003 in all cancers), including breast invasive carcinoma (BRCA), colon adenocarcinoma (COAD), head and neck squamous cell carcinoma (HNSC), kidney renal clear cell carcinoma (KIRC), kidney renal papillary cell carcinoma (KIRP), liver hepatocellular carcinoma (LIHC), lung adenocarcinoma (LUAD), lung squamous cell carcinoma (LUSC), prostate adenocarcinoma (PRAD), stomach adenocarcinoma (STAD), and uterine corpus endometrial carcinoma (UCEC) (Figures 1A–L). The other eight types of cancers with upregulated PRR11 include bladder urothelial carcinoma (BLCA), cervical squamous cell carcinoma and endocervical adenocarcinoma (CESC), cholangio carcinoma (CHOL), esophageal carcinoma (ESCA), glioblastoma multiforme (GBM), pheochromocytoma and paraganglioma (PCPG), rectum adenocarcinoma (READ), and sarcoma (SARC) (Supplementary Figure S2).

In addition, the high expression level of *PRR11* was associated with poor prognosis in 10 types of cancers, including adrenocortical carcinoma (ACC), BLCA, KICH, KIRP, brain lower grade glioma (LGG), LIHC, LUAD, mesothelioma (MESO), pancreatic adenocarcinoma (PAAD), and skin cutaneous melanoma (SKCM), which was identified using the Kaplan–Meier method and the log-rank test (Figures 1M–V). These findings demonstrated that PRR11 could play a pivotal role in pan-cancers development. Then we chose HepG2 (liver cancer cell line), NCI-H460 (lung cancer cell line), and BxPC3 (pancreatic cancer cell line) for the next experiment assay.

### Silencing PRR11 could inhibit cell proliferation and migration

**FIGURE 2:**
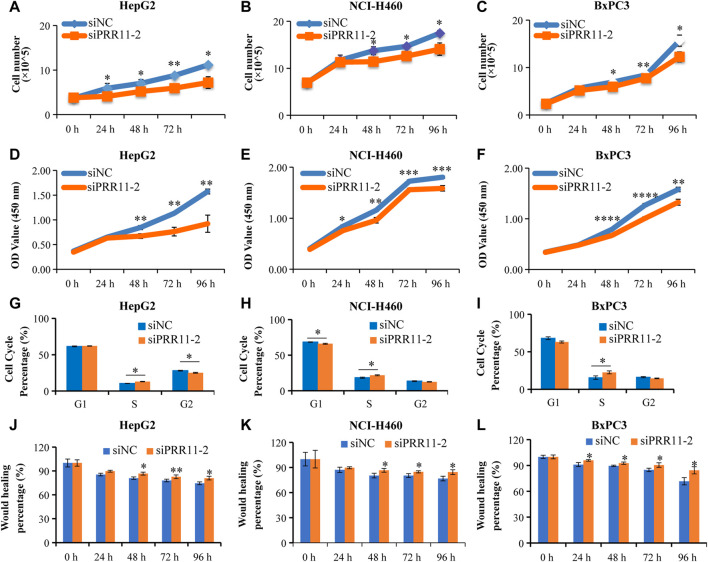
The low expression level of PRR11 strikingly inhibit tumor cell proliferation and migration. **(A-C)** Cell number of HepG2, NCI-H460, and BxPC3 after treating with siNC and siPRR11, respectively. **(D-F)** CCK-8 result of HepG2, NCI-H460, and BxPC3 after treating with siNC and siPRR11, respectively. **(G-I)** Cell cycle of HepG2, NCI-H460, and BxPC3 after treating with siNC and siPRR11, respectively. **(J-L)** Cell migration of HepG2, NCI-H460, and BxPC3 after treating with siNC and siPRR11, respectively. *p < 0.05, **p < 0.01, ***p < 0.001, and ****p < 0.0001.

HepG2, NCI-H460, and BxPC3 cells were treated with siPRR11-2 for 0, 24, 48, 72, and 96 h. From the results of cell counts and CCK-8 assay, a 24-h treatment with siPRR11-2 caused a significant decrease of cell proliferation in HepG2 (Figure 2A) and NCI-H460 (Figure 2E) (*p < 0.05) cell lines, whereas 48, 72, and 96-h treatment with siPRR11-2 caused a remarkable decrease of cell proliferation in all three kinds of cell lines (*p < 0.05, **p < 0.01, ***p < 0.001, and ****p < 0.0001) (Figures 2A–F). In addition, in all three kinds of tumor cells, siPRR11-2 treatment led to a significant arrest of the cell cycle to S phase (*p < 0.05, Figures 2G–I). Moreover, siPRR11-2 treatment significantly inhibited the cell migration of HepG2 (Figure 2J and Supplementary Figure S3A), NCI-H460 (Figure 2K and Supplementary Figure S3B), and BxPC3 (Figure 2L and Supplementary Figure S3C) compared with that of siNC at 48, 72, and 96 h (*p < 0.05 and **p < 0.01).

### PRR11 related DEGs and pathways identified by a bioinformatic analysis of TCGA pan-cancer data

First, we analyzed the DEGs with PRR11 high and low expression groups and found 1,320, 238, 327, 699, 1,127, 1944, 1,502, 362, 481, 841, and 1,127 DEGs in BRCA, COAD, HNSC, KIRC, KIRP, LIHC, LUAD, LUSC, STAD, PRAD, and UCEC, respectively (|FC| ≥ 1.5 and adjusted FDR < 0.05) (Supplementary Figure S4A). Among these 11 cancers in TCGA, 653 DEGs were screened out that occurred in more than four types of cancers (TCGA-4 Cs).

Then these 653 DEGs were analyzed by GO and KEGG analyses, and 10 enriched pathways of each analysis are shown in Supplementary Figure S5. The results indicated that DEGs were mainly enriched in the cell cycle pathway. For example, the GO_BP analysis showed that the DEGs were highly correlated to the cell cycle checkpoint, meiotic cell cycle, regulation of cell cycle G2/M phase transition, cell cycle G1/S phase transition, and DNA replication (Supplementary Figure S5A).

The KEGG result found that the DEGs were mainly enriched in the cell cycle, oocyte meiosis, complement and coagulation cascades, and IL-17 signaling pathway (Supplementary Figure S5B). The PPI analysis displayed that PTTG1 was highly correlated with cell division cycle 20 (CDC20), extra spindle pole bodies like 1, separase (ESPL1), cyclin-dependent kinase 1 (CDK1), aurora kinase A (AURKA), BUB1 mitotic checkpoint serine/threonine kinase (BUB1), BUB1 mitotic checkpoint serine/threonine kinase B (BUB1B), and cyclin B1 (CCNB1) with an interaction score ≥ 0.9 (Supplementary Figure S5C).

### PRR11 regulated the development of pan-cancer by influencing the expression of *PTTG1* gene and the cell cycle pathway by a bioinformatic analysis RNA-seq data of cell lines

**FIGURE 3:**
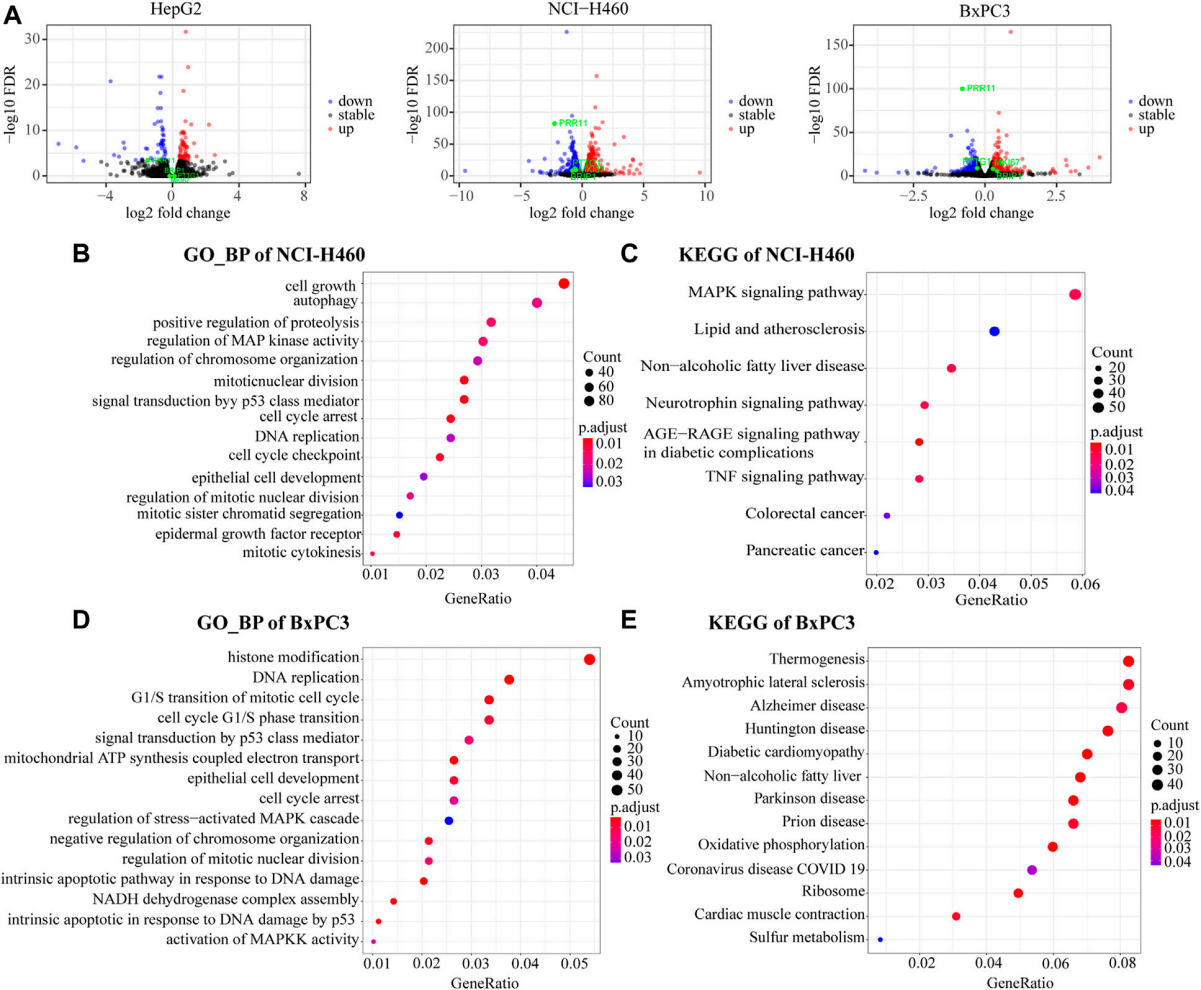
Functional enrichment analysis of RNA-Seq DEGs. **(A)** Volcano maps of HepG2 (left panel), NCI-H460 (middle panel), and BxPC3 (right panel). DEGs with threshold of |FC| > 1.2 and FDR < 0.1. *PRR11*, *PTTG1*, and *MKI67* were marked in green. **(B,C)** GO_BP and KEGG pathways of NCI-H460 DEGs. **(D,E)** GO_BP and KEGG pathway of BxPC3 DEGs.

We analyzed the DEGs in 48 h siNC and siPRR11 treatment groups of HepG2, NCI-H460, and BxPC3 cancer cells. As a result, a total of 119, 2,234, and 1,052 DEGs in HepG2, NCI-H460, and BxPC3 cancer cells, including 64, 1,398, and 577 up-DEGs and 55, 836, and 475 down-DEGs were screened with |FC| > 1.2 and FDR < 0.1 (Supplementary Figure S4A; Figure 3A). As a result, PTTG1 was a common DEG between siNC and siPRR11 treatment groups across three cell lines. Intriguingly, it was found that the expression levels of both PRR11 and PTTG1 were extremely correlated to genes involved in the cell cycle across various cancer types (Supplementary Figure S6). In addition, the significant enriched pathways of GO_BP pathways of HepG2 were response to alcohol, response to ketone, and response to mineralocorticoid and renal system process (*P*.adj <0.05) while no significant KEGG pathway (data not shown). In addition, the significant enriched pathways of GO_BP and KEGG for NCI-H460 and BxPC3 RNA-seq DEGs are shown in Figures 3B–E.

Collectively, the common significant GO_BP pathways for two out of three cell lines were cell cycle arrest, DNA replication, epithelial cell development, regulation of chromosome organization, regulation of mitotic nuclear division, MAPK signaling pathway, and signal transduction by the p53 class mediator.

### *PRR11* silencing could suppress the expression level of *PTTG1* in pan-cancer.

**FIGURE 4:**
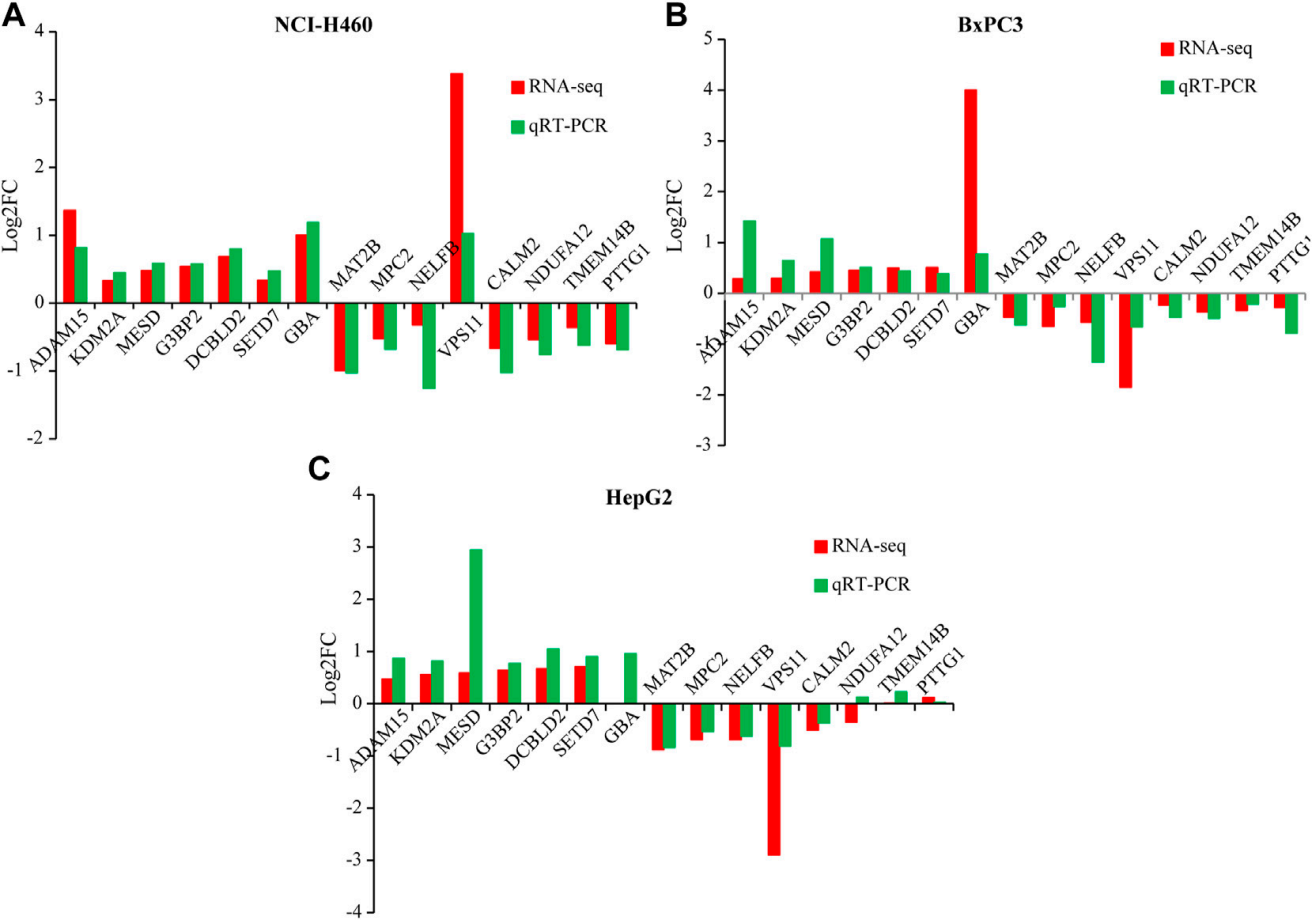
The qRT-PCR result verified the DEGs of RNA-seq from NCI-H460, BxPC3, and HepG2 with knocking down *PRR11*, respectively. **(A)** NCI-H460, **(B)** BxPC3, **(C)** HepG2.

**FIGURE 5:**
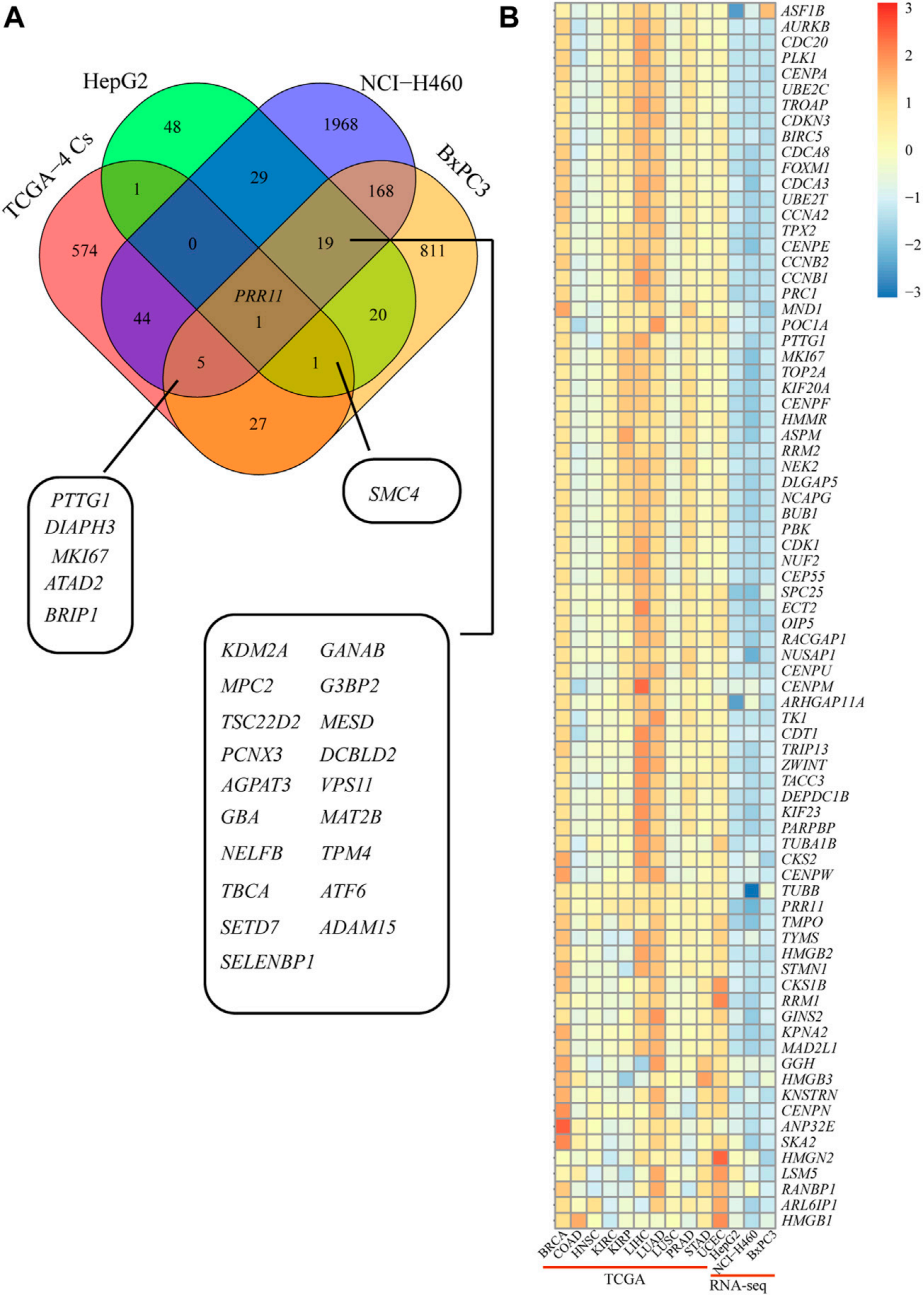
*PTTG1* might be a candidate downstream gene of *PRR11* and involved in the cell cycle. **(A)** Overlap DEGs between TCGA-4 Cs and RNA-seq. **(B)** Heat map of the cell cycle–related genes from both TCGA and RNA-seq. The colors represent the log2FC of different genes from 30% *PRR11* high and low group in BRCA, COAD, HNSC, KIRC, KIRP, LIHC, LUAD, LUSC, PRAD, STAD, and UCEC and between siPRR11 and siNC groups of HepG2, NCI-H460, and BxPC3 cancer cell lines.

In order to verify the reliability of the sequencing results, qRT-PCR was performed on 15 genes, including *ADAM15*, *KDM2A*, *MESD*, *G3BP2*, *DCBLD2*, *SETD7*, *GBA*, *MAT2B*, *MPC2*, *NELFB*, *VPS11*, *CALM2*, *NDUFA12*, *TMEM14B*, and *PTTG1* in HepG2, NCI-H460, and BxPC3 cells (Figures 4A–C and Figure 5A). Notably, *PRR11* was the only overlap gene among TCGA-4 Cs, HepG2, NCI-H460, and BxPC3 from RNA-seq DEGs at 48 h (Figure 5A). In addition, the heat map showed that the logFC value of cell cycle related genes between *PRR11* high and low groups. It was found that *PTTG1* involved in the cell cycle was upregulated in 10 out of 11 cancers and downregulated in NCI-H460 and BxPC3 cancer cell lines with interfering *PRR11* (Figure 5B). In addition, the rescue assay verified *PRR11*/*PTTG1* promoting cell proliferation in NCI-H460 (Supplementary Figure S4B). Thus, *PRR11* silencing in pan-cancers could suppress the expression level of *PTTG1*.

### PRR11 promoted the expression level of *PTTG1* by interacting with E2F1

**FIGURE 6:**
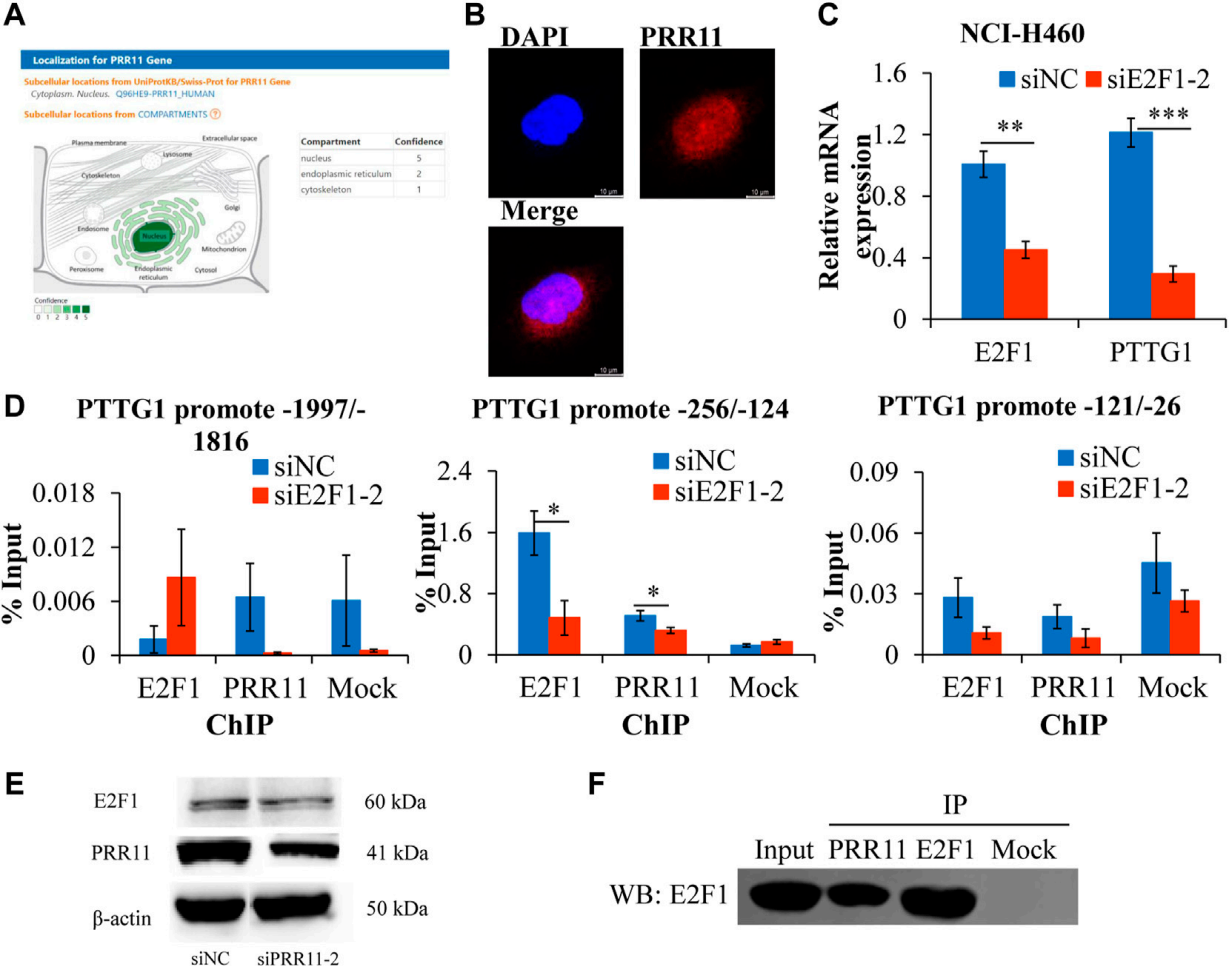
PRR11 promotes the expression level of PTTG1 by interacting with E2F1.**(A)** Localization for PRR11 gene was analyzed using GeneCards (https://www.genecards.org/). **(B)** Immunofluorescence localization of PRR11. **(C)** Expression levels of PTTG1 after interfering E2F1. **(D)** ChIP-qPCR of the *PTTG1* promoter region. **(E)** Expression levels of E2F1 and *PRR11* in NCI-H460 cells transfected with siNC or siPRR11 were examined by Western blotting. **(F)** Co-IP of *PRR11* and E2F1 in NCI-H460 cells. N = 3. *p < 0.05, **p < 0.01, and ***p < 0.001.

Given that *PTTG1* showed the same decreased expression trend in NCI-H460 and BxPC3 after knockdown of *PRR11* (Figure 3A, middle and right panels) and was involved in the cell cycle and sister chromatid separation events mentioned before, we speculated that PRR11 may be involved in the development of pan-cancer by regulating the expression of *PTTG1*. The Spearman correlation between *PRR11* and *PTTG1* was more than 0.3 in 16/33 types of cancer (with ≥200 samples) in TCGA (Supplementary Figure S7). Considering that the E2F1 transcription factor could enrich in the *PTTG1* promoter region to activate *PTTG1* expression (Wang et al., 2020) and PRR11 is located in the nucleus (using online website GeneCards^®^: The Human Gene Database, https://www.genecards.org/) (Figure 6A), we propose that PRR11 may regulate the expression level of *PTTG1* by combining with E2F1. Hence, we used the immunofluorescence localization assay to verify that PRR11 was located in the nucleus (Figure 6B). In addition, we used the JASPAR online tool to predict the E2F1 binding site on the *PTTG1* promoter region, followed by carrying out the qRT-PCR, ChIP-qPCR, and Co-IP assay when *E2F1* was knocked down and negative control for 48 h in NCI-H460 cells. As a result, the expression levels of the *PTTG1* gene was positively correlated with the expression level of *E2F1* (Figure 6C). In addition, the Western blot analysis indicated that knockdown of PRR11 inhibited the expression of E2F1 (Figure 6E), and the PRR11 could co-act with E2F1 and both of them could enrich in the E2F1 binding site (5′-TTTGGGGC-3′) of the *PTTG1* promoter region (−256/−124) *in vitro* (Figures 6D,F).

### *PTTG1* mRNA was highly expressed in pan-cancers and associated with poor overall survival of cancer patients

**FIGURE 7:**
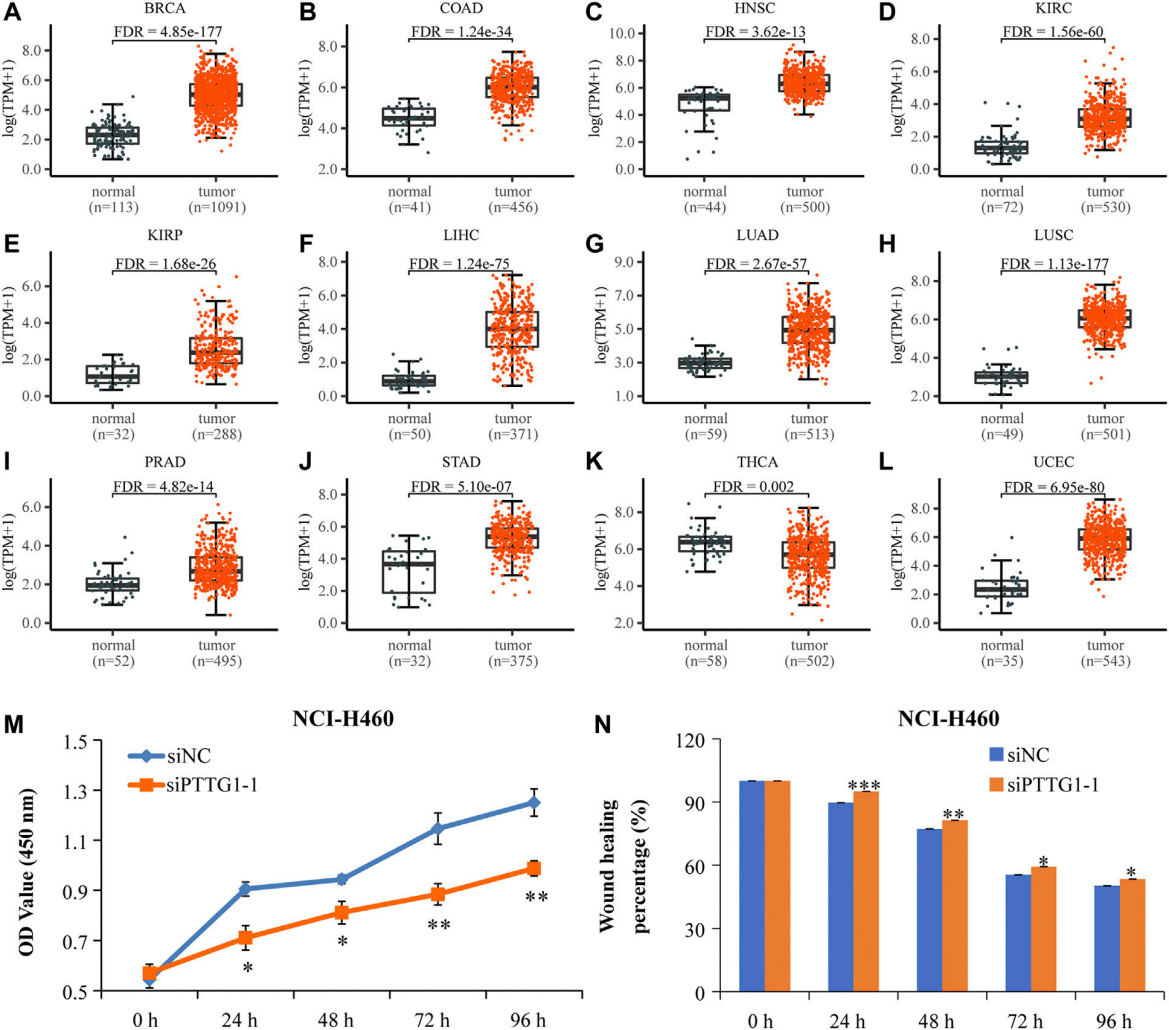
The high-expressed *PTTG1* in pan-cancers and interfering *PTTG1* could significantly suppress lung cancer cells’ proliferation and migration. **(A-L)** Scatter plots showing the expression levels of the *PTTG1* gene of normal tissues (black) and tumor tissues (red) in pan-cancer. **(M)** The low expression level of *PTTG1* strikingly repressed NCI-H460 cell proliferation. **(N)** The low-expressed *PTTG1* could significantly repress cell mobility of NCI-H460 cells. N = 6–9. *p < 0.05, **p < 0.01, and ***p < 0.001.

The expression level of *PTTG1* gene in pan-cancers is shown in Figure 7 and Supplementary Figure S6. Its expression in the tumor tissues was significantly higher than that in normal tissues, including BRCA, COAD, HNSC, KIRC, KIRP, LIHC, LUAD, LUSC, PRAD, STAD, and UCEC (Figures 7A–L). The other eight types of cancers with upregulated *PTTG1* include BLCA, CESC, CHOL, ESCA, GBM, KICH, PCPG, and READ (Supplementary Figures S8A–H). Moreover, the expression level of *PTTG1* was associated with poor prognosis in 10 kinds of cancers, including ACC, KIRC, KIRP, LGG LIHC, LUAD, MESO, PCPG, UCEC, and UVM (*p* ≤ 0.05) (Supplementary Figures S9A–J).

In addition, a GEO data set (GSE48075) verified that high expression of *PRR11* and *PTTG1* was significantly related to the poor prognosis of cancer patients (Supplementary Figures S8I,J). In addition, the Spearman correlation coefficient suggested a significant positive correlation between *PRR11* and *PTTG1* (Supplementary Figure S8K). All these results indicated that PTTG1 showed the same trends of expression and poor outcome as PRR11 and may be an oncogene in pan-cancer.

### *PTTG1* silencing significantly inhibited cell proliferation and migration of lung cancer cells

In order to observe the effect of *PTTG1* expression on cell proliferation and migration, NCI-H460 cells were treated with siPTTG1-1 for 0, 24, 48, 72, and 96 h. From the result of the CCK-8 assay, the 24- to 96-h treatment with siPTTG1-1 caused a dramatic decrease of cell proliferation in NCI-H460 (Figure 7M) (*p < 0.05, **p < 0.01). In addition, siPTTG1-1 treatments significantly repressed the cell migration of NCI-H460 (Figure 7N and Supplementary Figure S9K) compared with that of siNC at 24, 48, 72, and 96 h (*p <0.05, **p <0.01, and ***p <0.001). All these results implied that PTTG1 might play an important role in tumor development.

## Discussion

*PRR11* has been found highly expressed in various cancer types and strongly correlated with their poor outcome. In the present study, *PRR11* was highly expressed in 19 out of 33 kinds of cancers in TCGA. Our data have also shown that its upregulation was significant with poor outcome of various cancer patients. Recent evidence suggested that its upregulation may promote pan-cancer development by influencing cyclin genes and p38 MAPK (Lin et al., 2020), Wnt/β-catenin (Qiao et al., 2019; Zhou et al., 2019), PI3K/AKT/β-catenin (Zhu et al., 2018), and Akt/mTOR autophagy (Zhang et al., 2018) signaling pathways. Our findings confirmed that downregulated *PRR11* could inhibit the proliferation and migration of different cancer cells, which were consistent with previous results in the lung (Ji et al., 2013; Wang et al., 2015), pancreatic (Tan et al., 2017), and gastric (Song et al., 2015; Hu et al., 2018) cancer cells. Furthermore, our results also supported that inhibited *PRR11* could cause cell arrest in S phase, which is similar to that in lung cancer cells (Ji et al., 2013) and tongue squamous carcinoma (Wang et al., 2019).

In the current study, we performed RNA-seq in three tumor cell lines after interfering with *PRR11* for the first time. In order to thoroughly explore the cancer-promoting effects of *PRR11*, we integrated and analyzed the RNA-seq generated by ourselves and the TCGA pan-cancer data. Although it was reported that PRR11 could mediate the Akt/mTOR signaling pathway in non-small-cell lung cancer (Zhang et al., 2018), p38 MAPK signaling in pancreatic cancer cells (Lin et al., 2020), as well as Wnt/β-catenin signal transduction in both esophageal cancer (Zhou et al., 2019) and hepatic carcinoma (Qiao et al., 2019) to participate in cancer development, all of them do not significantly enrich in our GO and KEGG results of both TCGA and RNA-seq. For example, Zhang et al. (2018) showed that *PRR11* knockdown decreased AKT and mTOR phosphorylation levels. In contrast, RNA-seq analyses revealed the gene expression changes, but did not provide any information about the phosphorylation status of the AKT/mTOR pathway. Lin et al. (2019) showed that downregulation of *USP34* could inhibit proliferation and migration in pancreatic cancer cells and inactivate p38 MAPK signaling *via* inhibiting *PRR11*. It demonstrated that these signaling pathways are not common pathways for PRR11 to play a promotional role in pan-cancer. However, the cell cycle pathway was found in GO and KEGG results of both TCGA and RNA-seq. Furthermore, recent evidences (Ji et al., 2013; Zhang et al., 2015; Wang et al., 2019) and our results provided a strong case for *PRR11* regulation in S phase of the cell cycle in many cancers. These findings demonstrated that PRR11 might take part in development of pan-cancer by affecting genes involved in the cell cycle.

Unexpectedly, we did not find significant changes in the expression of cyclin genes with downregulation of *PRR11*, except cyclin D3 (*CCND3*) in BxPC3. Moreover, the early growth response protein 1 (*EGR1*) gene was also significantly suppressed after interfering *PRR11* in NCI-H460, which is consistent with the result of a previous study (Chen et al., 2015). From the result of the overlap genes between TCGA-4 Cs and RNA-seq, four genes were found to be influenced by *PRR11* downregulation, including *PTTG1*, *BRCA2*, *ATAD2*, and *BRIP1*. *PTTG1* has been reported as one of the cell cycle–related genes (Horning et al., 2018; Deng et al., 2021) and to be upregulated in breast, pituitary, ovarian, and uterine cancers (Vlotides et al., 2007). Previous research works have shown that high *PTTG1* expression could enhance breast cancer malignancy by augmenting breast cancer stem cell population and epithelial mesenchymal transition in a manner dependent on activation of the PI3K/AKT pathway (Yoon et al., 2012). In the current study, to gain mechanistic insights of how *PRR11* regulates the expression of *PTTG1*, we used ChIP-qPCR and Co-IP to prove that *PRR11* was expressed in the nucleus and can interact with and be recruited by E2F1 to the E2F1 binding site (5′-TTTGGGGC-3′) of the *PTTG1* promoter region (−256/−142), consequently stimulate the expression of *PTTG1*. This study provided new ideas for future exploration of the function of *PRR11* in promoting tumor development. Moreover, the novel role of PRR11 in modulating *PTTG1* expression showing its positive function in the cell cycle will likely encourage people to study the functions of PRR11 in the development of pan-cancer.

In summary, our results revealed that PRR11 affect cancer development through cell apoptosis and autophagy, DNA replication, nuclear division, sister chromatid separation, MAPK signal pathway, and signal transduction by p53 class mediator signal pathways. Notably, PRR11 may play an oncogenic role in pan-cancer progression by regulating the expression of *PTTG1* to involve in the cell cycle. In mechanism, PRR11 could combine with E2F1 to the E2F1 binding site of the *PTTG1* promoter region, which consequently could stimulate the expression of *PTTG1*. To our knowledge, this is the first study demonstrating the common regulation role of *PRR11* on *PTTG1* in different human cancer cells.

## Data Availability

The datasets presented in this study can be found in online repositories. The names of the repository/repositories and accession number(s) can be found in the article/Supplementary Material.

## Abbreviations

TCGA-4 Cs, more than four types of cancers in 11 cancers with normal tissues ≥ 30 in TCGA.
