# Supplementary material for: PRR11 promotes cell proliferation by regulating PTTG1 through interacting with E2F1 transcription factor in pan-cancer
Source: Front Mol Biosci. 2022 Aug 19;9:877320. doi: 10.3389/fmolb.2022.877320 (PMC9437250; doi:10.3389/fmolb.2022.877320)
Supplement: Supplementary file 1 [file DataSheet3.docx]

**Table S1. The small interfering RNA for PRR11 in this study**

| **Name** | **Strand** | **Sequence** | **Application** |
| --- | --- | --- | --- |
| siPRR11-1 | sense | CCAGAAAGAGUCGGUAUUUTT | Interference PRR11 |
|  | antisense | AAAUACCGACUCUUUCUGGTT |  |
| siPRR11-2 | sense | CCAUCUUUCCAUCUCGUAUTT |  |
|  | antisense | AUACGAGAUGGAAAGAUGGTT |  |
| siPRR11-3 | sense | GGAUCUGCGGAAACUGCUUTT |  |
|  | antisense | AAGCAGUUUCCGCAGAUCCTT |  |
| siE2F1-1 | sense | GCUAUGAGACCUCACUGAATT | Interference E2F1 |
|  | antisense | UUCAGUGAGGUCUCAUAGCTT |  |
| siE2F1-2 | sense | GCAUCUAUGACAUCACCAATT |  |
|  | antisense | UUGGUGAUGUCAUAGAUGCTT |  |
| siE2F1-3 | sense | AGAUGGUUAUGGUGAUCAATT |  |
|  | antisense | UUGAUCACCAUAACCAUCUTT |  |
| siPTTG1-2 | sense | GGGAGAUCUCAAGUUUCAATT | Interference PTTG1 |
|  | antisense | UUGAAACUUGAGAUCUCCCTT |  |
| siNC | sense | UUCUCCGAACGUGUCACGUTT | Interference negative control |
|  | antisense | ACGUGACACGUUCGGAGAATT |  |

**Table S2. The primers used in this study**

| **Name** | **Strand** | **Sequence** | **Application** |
| --- | --- | --- | --- |
| PRR11 | sense | CGTATCTGCCACCGAGAACTT | Quantitative real-time PCR (qRT-PCR) |
|  | antisense | GAGATGGTCTTCAGTGCTTCCT |  |
| E2F1 | sense | CCAUCUUUCCAUCUCGUAUTT |  |
|  | antisense | AUACGAGAUGGAAAGAUGGTT |  |
| PTTG1 | sense | ACCCGTGTGGTTGCTAAGG |  |
|  | antisense | ACGTGGTGTTGAAACTTGAGAT |  |
| ADAM15 | sense | AAATACCGGGACTTCCAGCA |  |
|  | antisense | AGTGCCACTCGTACATTCAGG |  |
| KDM2A | sense | TCTGTCTTCCGCTACCTCA |  |
|  | antisense | GCACAATGGCCTTACACC |  |
| MESD | sense | TTGCCCTCCCAAGTGGTT |  |
|  | antisense | TGTACGCAGCCTCTCCAGT |  |
| G3BP2 | sense | TCAGCGACTCTTCTGACCT |  |
|  | antisense | CTGGGCTTCTCCATAACCAT |  |
| DCBLD2 | sense | CAGCAGCGAAGTTAATCACC |  |
|  | antisense | TCCTACCAGTGGCTGAGCAT |  |
| SETD7 | sense | CATTTGACATAAGACAGCGTGA |  |
|  | antisense | CTAATCATTTGGCATCTCCGA |  |
| GBA | sense | CTTGCCCTGAACCCCGAA |  |
|  | antisense | TCGTTCTTCTGACTGGCAAC |  |
| MAT2B | sense | CCCATCCCGTATCTGCTCT |  |
|  | antisense | GGCACCAGTAACCAGAACC |  |
| MPC2 | sense | GGCTGATATGGCCAGACC |  |
|  | antisense | CCCCACAAAGAAATTAACAGCA |  |
| NELFB | sense | CCATCGAGCAGTTCCAGACA |  |
|  | antisense | GCAGCTCATCGAATACCGACT |  |
| VPS11 | sense | GACCGGAAGGTTTCTCCCAA |  |
|  | antisense | CATCCTCAAAGACGGTGCTA |  |
| CALM2 | sense | ATGGTAATGGCACAATTGACT |  |
|  | antisense | ATCAAACACACGGAATGCTT |  |
| NDUFA12 | sense | CCTCCTGAATGGCATCGTT |  |
|  | antisense | CGTCCAAATGAATTTACGAGCA |  |
| TMEM14B | sense | CCTCTTCCCATTAGTGCCTT |  |
|  | antisense | CATAGCCAACGATCCCACCA |  |
| GAPDH | sense | ACAACTTTGGTATCGTGGAAGG |  |
|  | antisense | GCCATCACGCCACAGTTTC |  |
| PTTG1-P1 | sense | CCTGCCTCAATAAAATAGCC | ChIP-qPCR for PTTG1 promoter |
|  | antisense | AAAAACAAAGCCTGAGTGAT |  |
| PTTG1-P2 | sense | ATGCAGCTTAATAATATGGAGAG |  |
|  | antisense | GAAAGGACCTAAGCAGCCAG |  |
| PTTG1-P3 | sense | GCTGCTTAGGTCCTTTCCAT |  |
|  | antisense | CGGCGCACTCCTGGTTT |  |
