# Supplementary figures and images for: PRR11 promotes cell proliferation by regulating PTTG1 through interacting with E2F1 transcription factor in pan-cancer
Source: Front Mol Biosci. 2022 Aug 19;9:877320. doi: 10.3389/fmolb.2022.877320 (PMC9437250; doi:10.3389/fmolb.2022.877320)

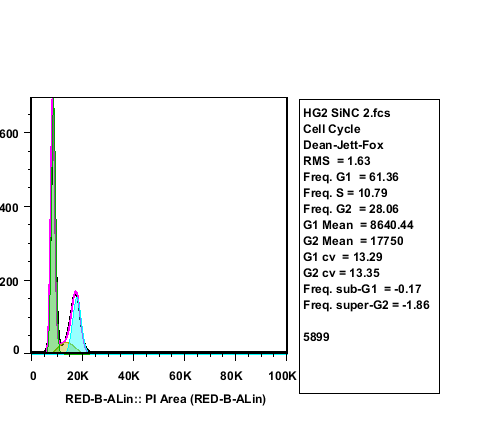

Supplement: Supplementary file 2 [file DataSheet4.zip › Figure 2/Figure 2G HepG2/HG2 SiNC 2.png]

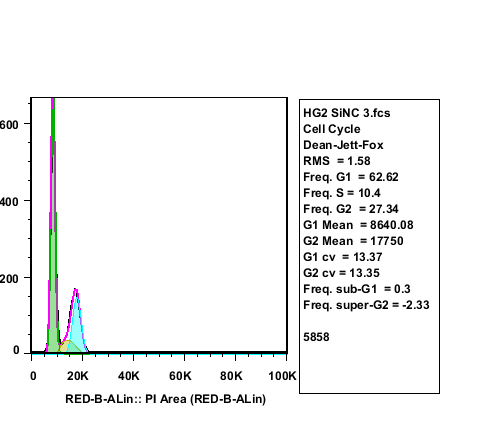

Supplement: Supplementary file 2 [file DataSheet4.zip › Figure 2/Figure 2G HepG2/HG2 SiNC 3.png]

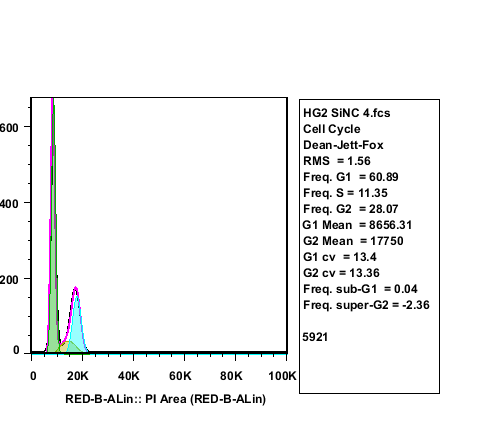

Supplement: Supplementary file 2 [file DataSheet4.zip › Figure 2/Figure 2G HepG2/HG2 SiNC 4.png]

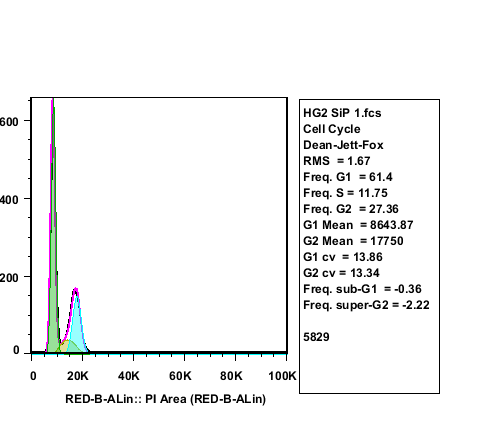

Supplement: Supplementary file 2 [file DataSheet4.zip › Figure 2/Figure 2G HepG2/HG2 SiP 1.png]

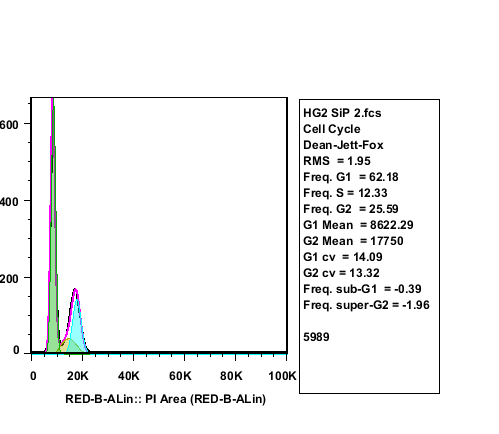

Supplement: Supplementary file 2 [file DataSheet4.zip › Figure 2/Figure 2G HepG2/HG2 SiP 2.png]

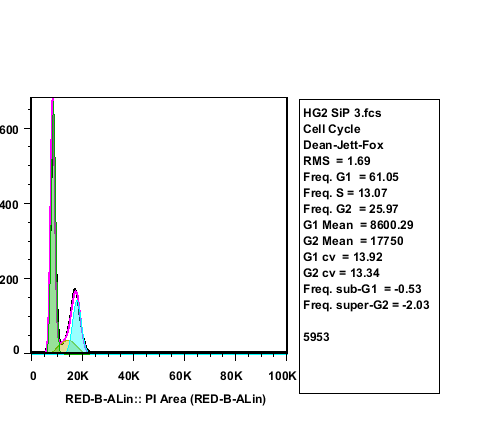

Supplement: Supplementary file 2 [file DataSheet4.zip › Figure 2/Figure 2G HepG2/HG2 SiP 3.png]

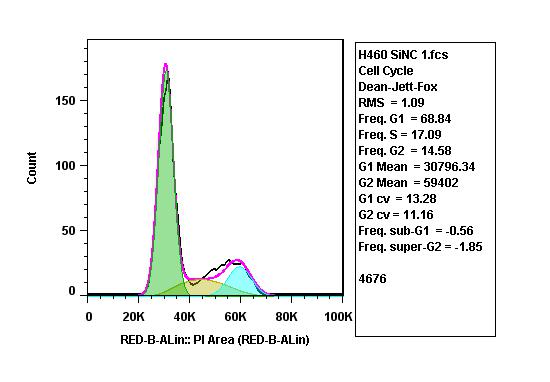

Supplement: Supplementary file 2 [file DataSheet4.zip › Figure 2/Figure 2H NCI-H460/H460 SiNC 1.jpg]

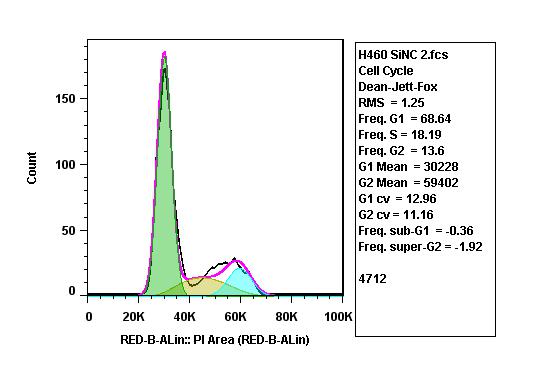

Supplement: Supplementary file 2 [file DataSheet4.zip › Figure 2/Figure 2H NCI-H460/H460 SiNC 2.jpg]

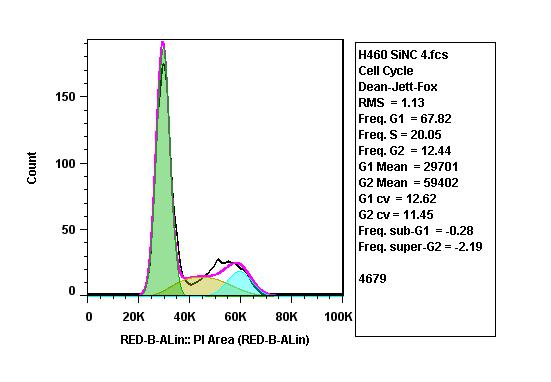

Supplement: Supplementary file 2 [file DataSheet4.zip › Figure 2/Figure 2H NCI-H460/H460 SiNC 4.jpg]

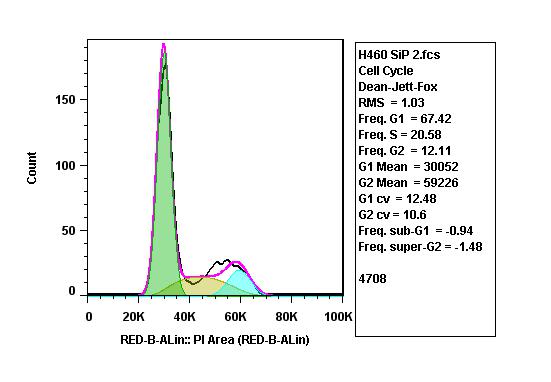

Supplement: Supplementary file 2 [file DataSheet4.zip › Figure 2/Figure 2H NCI-H460/H460 SiP 2.jpg]

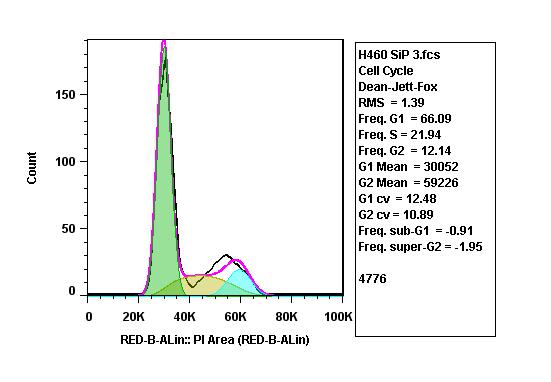

Supplement: Supplementary file 2 [file DataSheet4.zip › Figure 2/Figure 2H NCI-H460/H460 SiP 3.jpg]

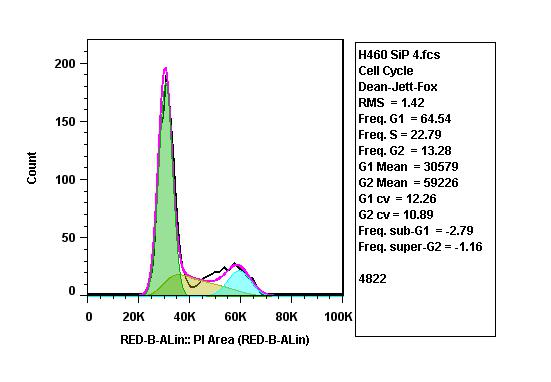

Supplement: Supplementary file 2 [file DataSheet4.zip › Figure 2/Figure 2H NCI-H460/H460 SiP 4.jpg]

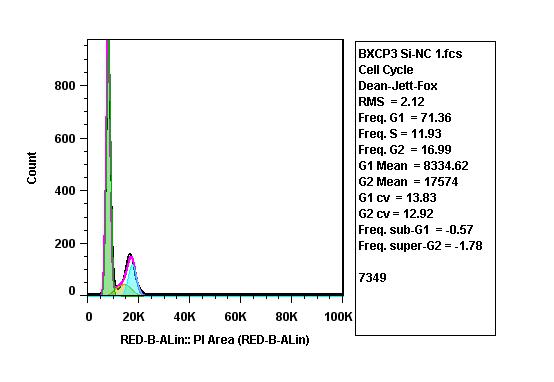

Supplement: Supplementary file 2 [file DataSheet4.zip › Figure 2/Figure 2I BxPC3/BXCP3 Si-NC 1.jpg]

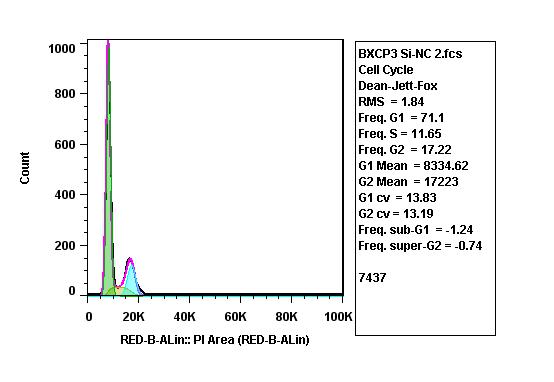

Supplement: Supplementary file 2 [file DataSheet4.zip › Figure 2/Figure 2I BxPC3/BXCP3 Si-NC 2.jpg]

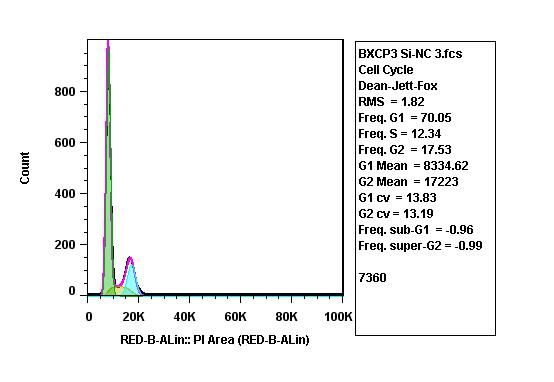

Supplement: Supplementary file 2 [file DataSheet4.zip › Figure 2/Figure 2I BxPC3/BXCP3 Si-NC 3.jpg]

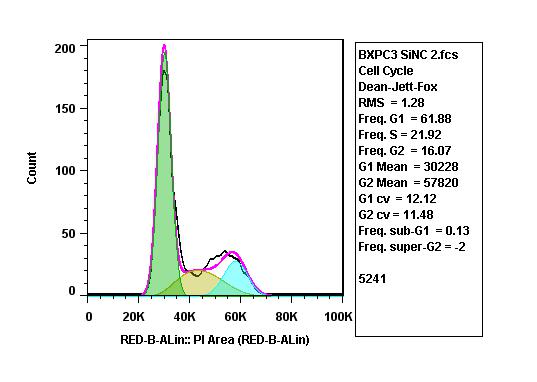

Supplement: Supplementary file 2 [file DataSheet4.zip › Figure 2/Figure 2I BxPC3/BXPC3 SiNC 2.jpg]

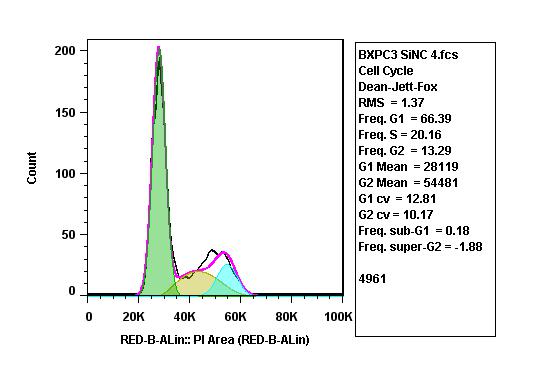

Supplement: Supplementary file 2 [file DataSheet4.zip › Figure 2/Figure 2I BxPC3/BXPC3 SiNC 4.jpg]

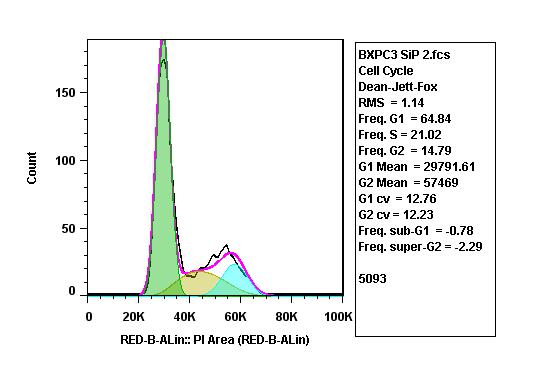

Supplement: Supplementary file 2 [file DataSheet4.zip › Figure 2/Figure 2I BxPC3/BXPC3 SiP 2.jpg]

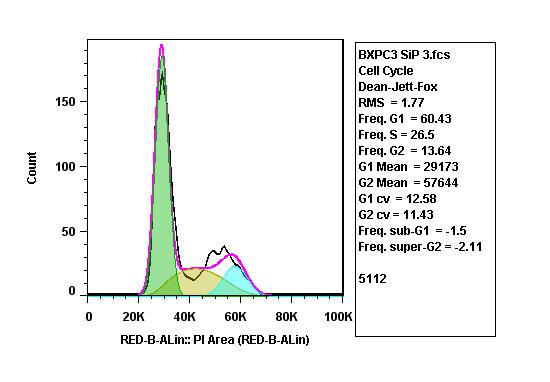

Supplement: Supplementary file 2 [file DataSheet4.zip › Figure 2/Figure 2I BxPC3/BXPC3 SiP 3.jpg]

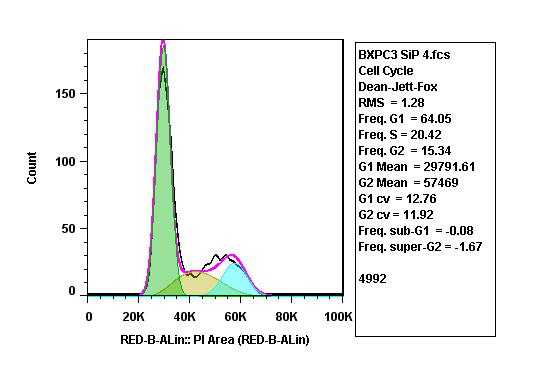

Supplement: Supplementary file 2 [file DataSheet4.zip › Figure 2/Figure 2I BxPC3/BXPC3 SiP 4.jpg]

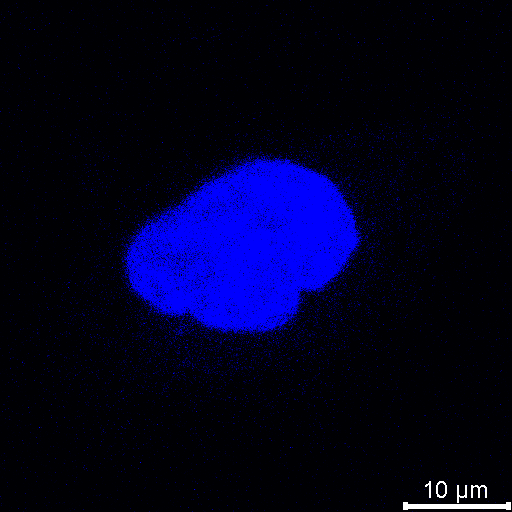

Supplement: Supplementary file 4 [file DataSheet2.ZIP › Figure 6/Figure 6B DAPI.tif]

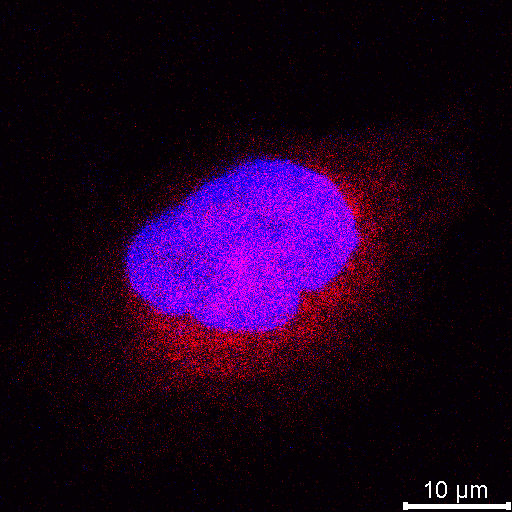

Supplement: Supplementary file 4 [file DataSheet2.ZIP › Figure 6/Figure 6B Merge.tif]

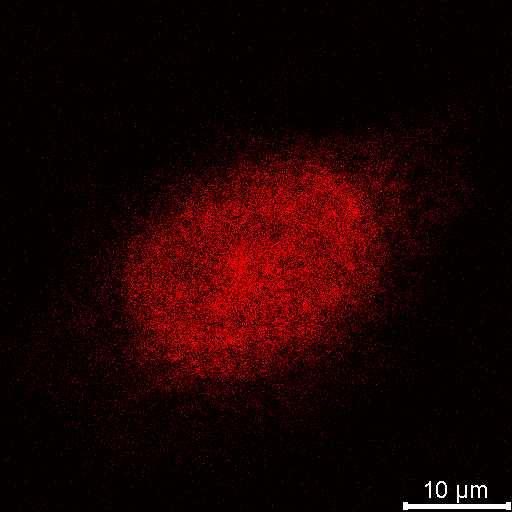

Supplement: Supplementary file 4 [file DataSheet2.ZIP › Figure 6/Figure 6B PRR11.tif]

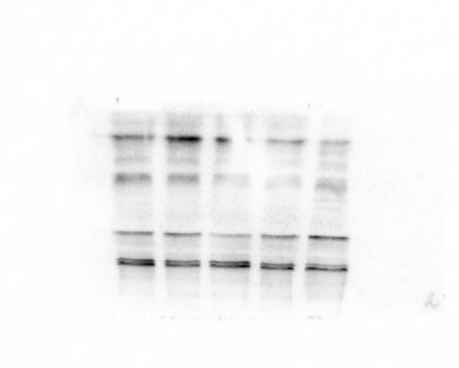

Supplement: Supplementary file 4 [file DataSheet2.ZIP › Figure 6/Figure 6E E2F1 Antibody.png]

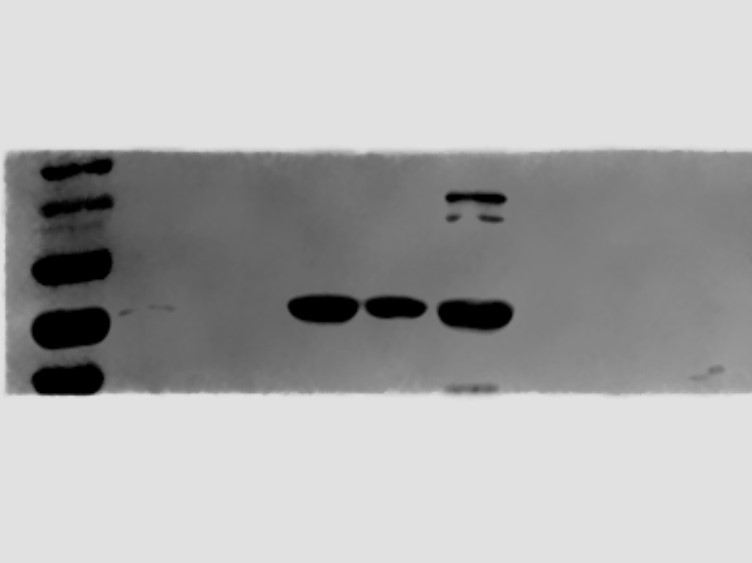

Supplement: Supplementary file 4 [file DataSheet2.ZIP › Figure 6/Figure 6E E2F1 Antibody.tif]

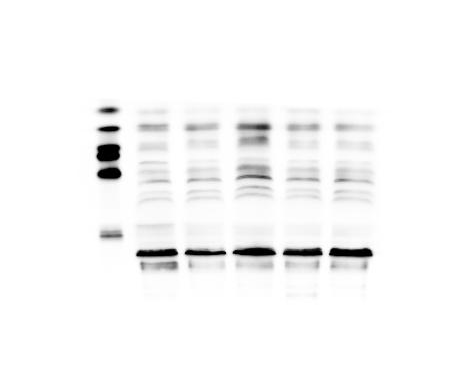

Supplement: Supplementary file 4 [file DataSheet2.ZIP › Figure 6/Figure 6E PRR11 Antibody.png]

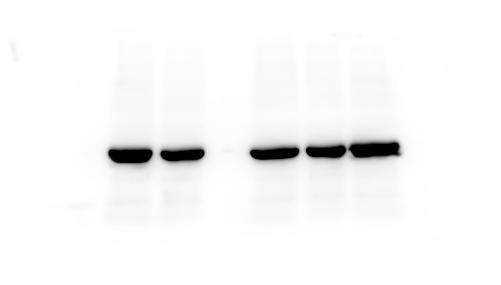

Supplement: Supplementary file 4 [file DataSheet2.ZIP › Figure 6/Figure 6E a┬-actin antibody.png]
