# Supplementary material for: PRR11 promotes cell proliferation by regulating PTTG1 through interacting with E2F1 transcription factor in pan-cancer
Source: Front Mol Biosci. 2022 Aug 19;9:877320. doi: 10.3389/fmolb.2022.877320 (PMC9437250; doi:10.3389/fmolb.2022.877320)
Supplement: Supplementary file 3 [file DataSheet1.PDF]

Supplementary Figures

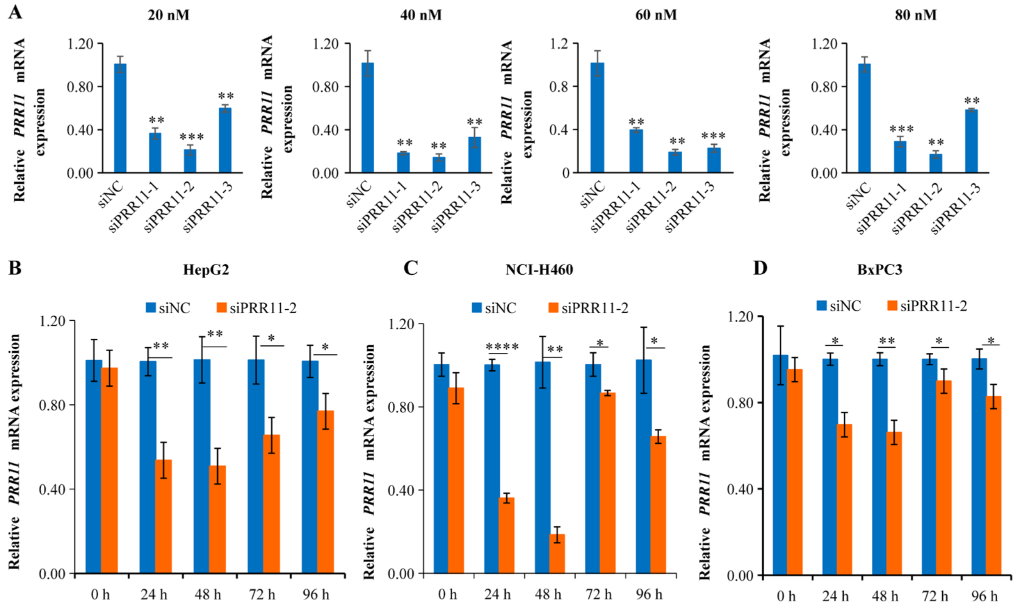

Supplementary Figure 1. The PRR11 mRNA expression was determined in HepG2, NCI-H460 and BxPC3 cell lines after interfering PRR11 using qRT-PCR. **(A)** The PRR11 mRNA expression was determined in HepG2 after interfering with different concentrations of small interfering RNA of PRR11. **(B-D)** The PRR11 mRNA expression was detected in HepG2, NCI-H460 and BxPC3 cell lines after interfering PRR11 using 40 nM siPRR11-2 at different time points, respectively. Graphs are representative of two independent experiments, each performed in triplicate. Error bars represent ± SEM of triplicate values. Statistical significance was determined by Student's t-test (*$P < 0.05$, **$P < 0.01$, ***$P < 0.001$, ****$P < 0.0001$).

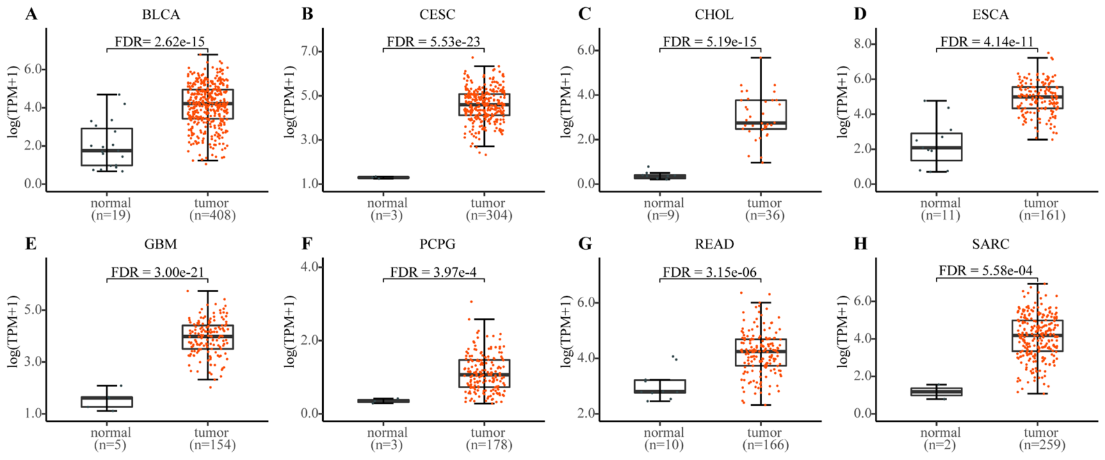

Supplementary Figure 2. The expression level of PRR11 of normal tissues (black) and tumor tissues (red) in pan-cancer with normal tissues less than 30**. (A)** BLCA, bladder

urothelial carcinoma; **(B)** CESC, cervical squamous cell carcinoma and endocervical adenocarcinoma; **(C)** CHOL, cholangio carcinoma; **(D)** ESCA, esophageal carcinoma; **(E)** GBM, glioblastoma multiforme; **(F)** PCPG, pheochromocytoma and paraganglioma; **(G)** READ, and rectum adenocarcinoma; **(H)** SARC, sarcoma.

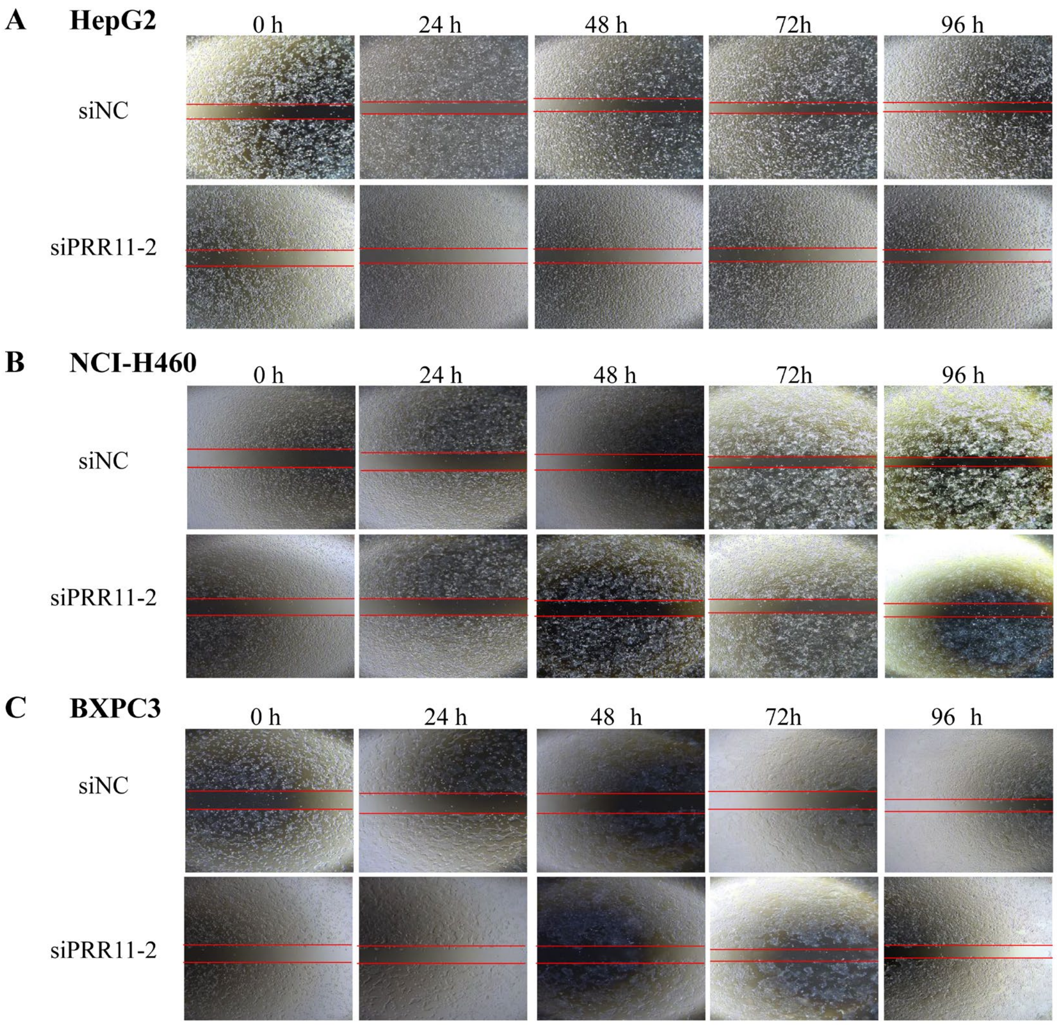

Supplementary Figure 3. The wound healing distance after interfering PRR11 in different cancer cell lines. **(A-C)** The wound distance at 0, 24, 48, 72 and 96 h of HepG2, NCI-H460 and BxPC3 after treating with siNC and siPRR11, respectively.

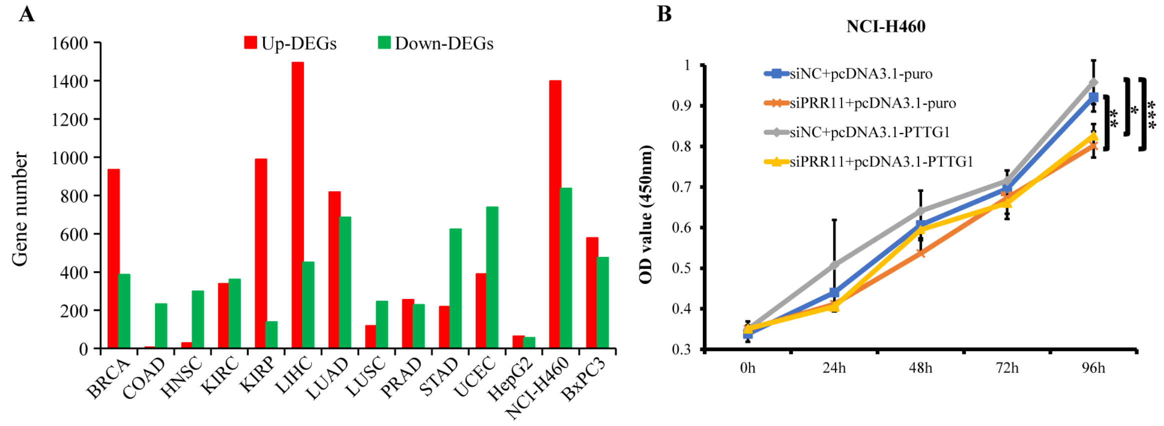

Supplementary Figure 4. Validating RNA-seq results with qRT-PCR. **(A)** The number of DEGs in different cancers of TCGA and RNA-seq. **(B)** After interference with *PRR11*, overexpressed *PTTG1*, and then used CCK-8 to detect cell proliferation. N=3, **P* < 0.05; ***P* < 0.01; ****P* < 0.001; *****P* < 0.0001.

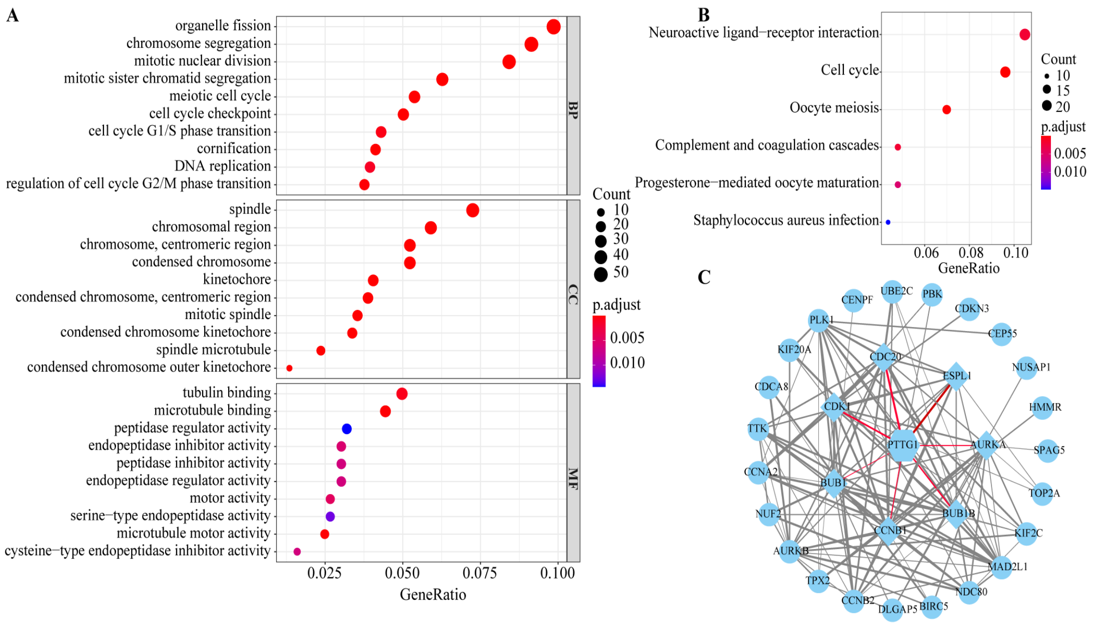

Supplementary Figure 5. The GO and KEGG results of DEGs of TCGA-4 Cs**. (A and B)** The top 10 pathways of GO_BP, GO_CC, GO_MF and KEGG of TCGA-4 Cs, respectively. **(C)** The PPI result of TCGA-4 Cs with interaction score ≥ 0.9 from interaction sources of textmining and experiments.

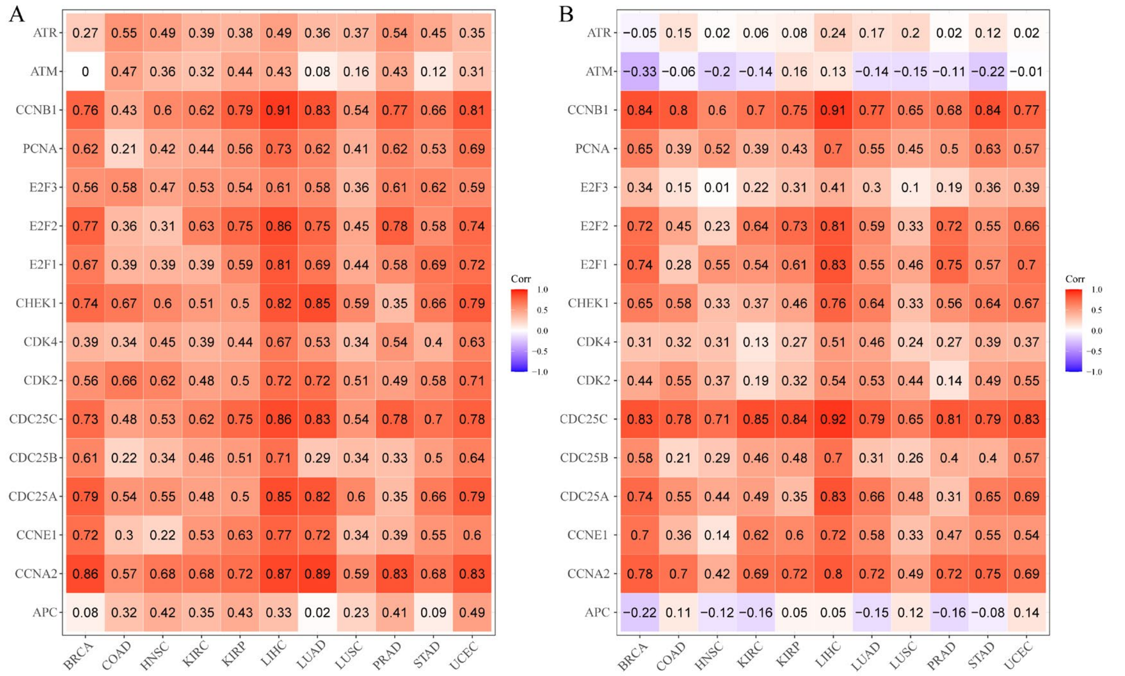

Supplementary Figure 6. The expression of both PRR11 and PTTG1 were extremely correlated to cell cycle mark genes in 11 types of cancer from TCGA database. **(A)** the correlation between the expression of PRR11 and cell cycle mark genes in different cancers. **(B)** the correlation between the expression of PTTG1 and cell cycle mark genes in different cancers.

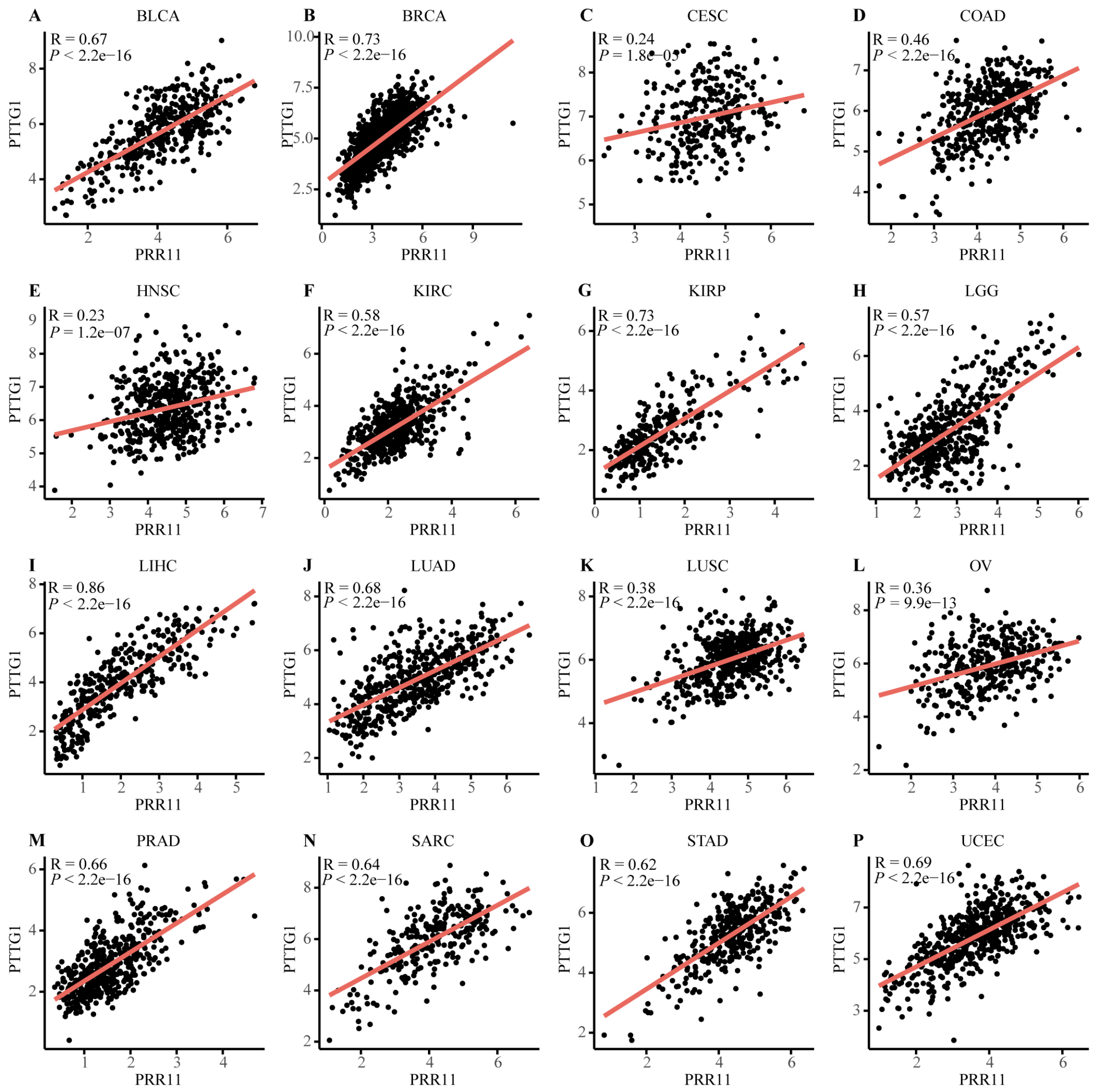

Supplementary Figure 7. The spearman correlation between PRR11 and PTTG1 in pan-cancer. **(A)** BLCA, bladder urothelial carcinoma; **(B)** BRCA, Breast invasive carcinoma; **(C)** CESC, cervical squamous cell carcinoma and endocervical adenocarcinoma; **(D)** COAD, Colon adenocarcinoma; **(E)** HNSC, Head and Neck squamous cell carcinoma; **(F)** KIRC, Kidney renal clear cell carcinoma; **(G)** KIRP, Kidney renal papillary cell carcinoma; **(H)** LGG, brain lower grade glioma; **(I)** LIHC, Liver hepatocellular carcinoma; **(J)** LUAD, Lung adenocarcinoma; **(K)** LUSC, lung squamous cell carcinoma; **(L)** OV, Ovarian cancer; **(M)** PRAD, Lung squamous cell carcinoma Prostate adenocarcinoma; **(N)** SARC, Sarcoma; **(O)** STAD, Stomach adenocarcinoma; **(P)** UCEC, Uterine Corpus Endometrial Carcinoma.

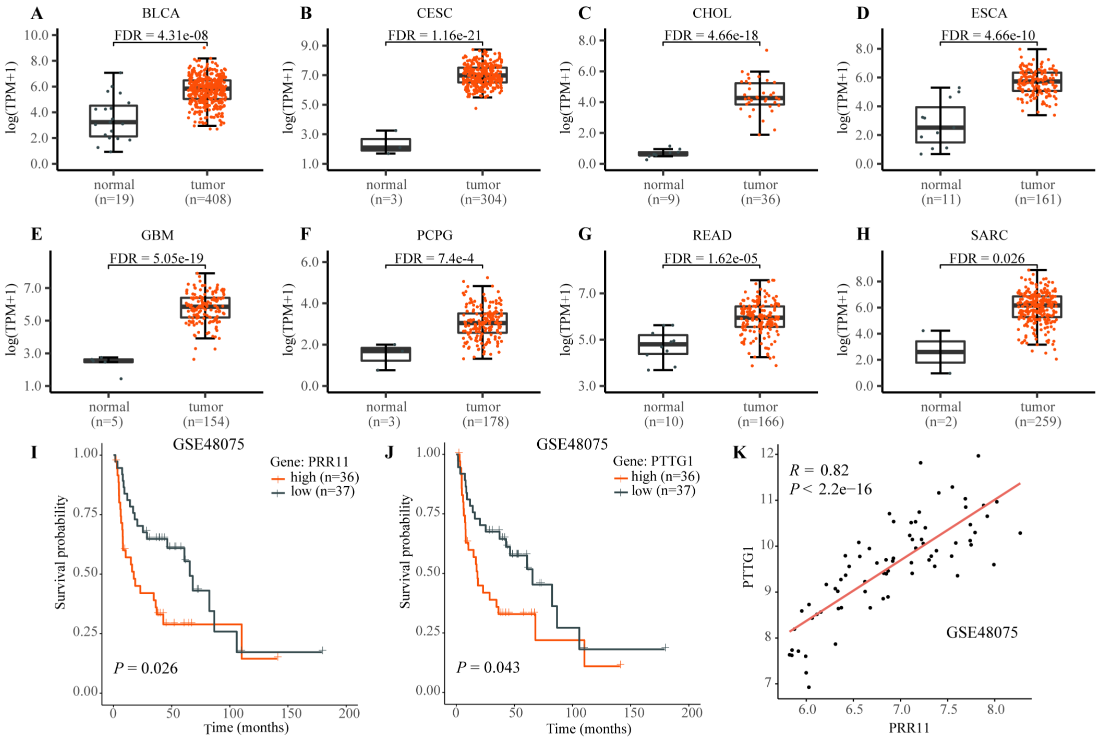

Supplementary Figure 8. The high-expressed PTTG1 in different cancer types with normal tissues less than 30. **(A)** BLCA, bladder urothelial carcinoma; **(B)** CESC, cervical squamous cell carcinoma and endocervical adenocarcinoma; **(C)** CHOL, cholangio carcinoma; **(D)** ESCA, esophageal carcinoma; **(E)** GBM, glioblastoma multiforme; **(F)** PCPG, pheochromocytoma and paraganglioma; **(G)** READ, rectum adenocarcinoma; **(H)** SARC, sarcoma. **(I-J)** The relationship between the expression levels of PRR11, PTTG1 and the patients OS in GSE48075, respectively. **(K)** The spearman correlation coefficient between PRR11 and PTTG1 in GSE48075.

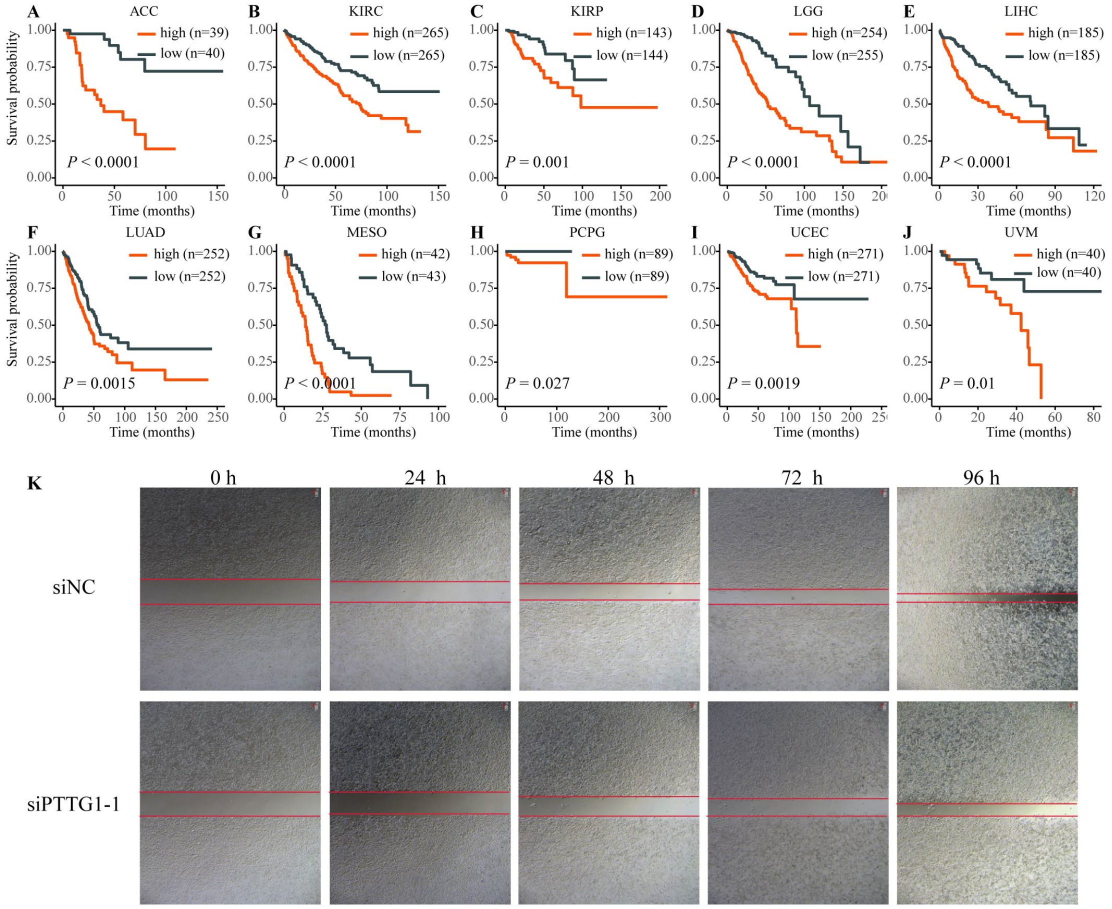

Supplementary Figure 9. The high-expressed *PTTG1* significantly associated with overall survival. **(A-J)** Analysis of the role of *PTTG1* gene in the overall survival in different kinds of cancer patients. **(K)** The wound distance at 0, 24, 48, 72 and 96 h of NCI-H460 after treating with siNC and siPTTG-1, respectively.
